# Multielement Z-tag imaging by X-ray fluorescence microscopy for next-generation multiplex imaging

**DOI:** 10.1038/s41592-023-01977-x

**Published:** 2023-08-31

**Authors:** Merrick Strotton, Tsuyoshi Hosogane, Marco di Michiel, Holger Moch, Zsuzsanna Varga, Bernd Bodenmiller

**Affiliations:** 1 Department of Quantitative Biomedicine, University of Zurich, Zurich, Switzerland; 2 Institute for Molecular Health Sciences, ETH Zurich, Zurich, Switzerland; 3 Life Science Zurich Graduate School, ETH Zurich and University of Zurich, Zurich, Switzerland; 4 ESRF, The European Synchrotron, Grenoble, France; 5 Department of Pathology and Molecular Pathology, University Hospital Zurich, Zurich, Switzerland

**Keywords:** Molecular imaging, Cancer imaging

## Abstract

Rapid, highly multiplexed, nondestructive imaging that spans the molecular to the supra-cellular scale would be a powerful tool for tissue analysis. However, the physical constraints of established imaging methods limit the simultaneous improvement of these parameters. Whole-organism to atomic-level imaging is possible with tissue-penetrant, picometer-wavelength X-rays. To enable highly multiplexed X-ray imaging, we developed multielement Z-tag X-ray fluorescence (MEZ-XRF) that can operate at kHz speeds when combined with signal amplification by exchange reaction (SABER)-amplified Z-tag reagents. We demonstrated parallel imaging of 20 Z-tag or SABER Z-tag reagents at subcellular resolution in cell lines and multiple human tissues. We benchmarked MEZ-XRF against imaging mass cytometry and demonstrated the nondestructive multiscale repeat imaging capabilities of MEZ-XRF with rapid tissue overview scans, followed by slower, more sensitive imaging of low-abundance markers such as immune checkpoint proteins. The unique multiscale, nondestructive nature of MEZ-XRF, combined with SABER Z-tags for high sensitivity or enhanced speed, enables highly multiplexed bioimaging across biological scales.

## Abstract (web summary)

Multielement Z-tag X-ray fluorescence (MEZ-XRF) offers a new avenue for nondestructive and highly multiplexed tissue imaging and operates from the nanometer to whole-tissue scale, unlocking new biological observations.

## Main

Imaging multiple molecular features across different spatial scales can reveal how function emerges from complex biological systems. At the tissue level, imaging of molecular markers facilitates characterization of cell phenotypes and states in relation to tissue structure, whereas subcellular marker localization (for example, whether a receptor is internalized) can shed light on cellular mechanisms (for example, response to stimulus). Most bioimaging methods are based on imaging probes (for example, laser, ultraviolet or ion beam) and molecular tags (for example, fluorescent, mass, oligonucleotide tags) that have inherent speed, depth and spatial resolution limits at key biological scales. Further, most methods cause tissue and/or reagent destruction, preventing repeat measurement of multiple molecular markers or subsequent sample analysis. Imaging methods often maximize one or two parameters at the expense of others. For instance, electron microscopy offers subnanometer ultrastructure imaging but at relatively low speed and throughput with limited multiplexing^1^. Conversely, MALDI-based imaging can characterize thousands of tissue analytes in an unbiased way, although with low-micrometer spatial resolution and low sensitivity^2^.

For fluorescent imaging, spectral overlap limits the number of fluorescent tags that can be imaged in parallel. The parallel imaging issue has been circumvented with iterative staining^3–6^ and nucleotide-based barcoding^7,8^ approaches that enable imaging of tens or hundreds of tags, respectively, potentially with signal amplification with techniques such as signal amplification by exchange reaction (SABER)^8^. However, these approaches have practical limitations. Iterative staining is slow and destructive to epitopes and tissues, limiting the number of times a region of interest (ROI) can be probed. For highly multiplexed barcode-based approaches, high-resolution images may be necessary to spatially separate barcodes, which limits throughput and prevents low-resolution imaging to guide ROI selection. The diffraction limit has been overcome with super-resolution^9^ and expansion microscopy^10^ approaches, but these methods suffer from low throughput. Moreover, autofluorescence limits use of fluorescent-tag approaches in formalin-fixed, paraffin embedded (FFPE) clinical samples. In short, the physical constraints of fluorescent microscopy complicate the parallel optimization of multiple microscopy parameters^11^.

Some fluorescent imaging limits can be circumvented by using other molecular tags. For instance, imaging mass cytometry (IMC)^12^ and multiplexed ion beam imaging (MIBI)^13^ rely on mass tags allowing analysis of 40 or more markers simultaneously. Alternatively, spatially encoded, nucleotide tags can be liberated from samples and sequenced to profile at 1,000-plex or more^14,15^. However, these approaches are restricted to two-dimensional (2D) imaging of exposed surfaces and can thus only be applied in three dimensions by analysis of serial sections^16^. With the exception of MIBI^17^, these techniques are destructive, precluding repeat tissue analysis. Nucleotide tags can be imaged via fluorescence enabling three-dimensional (3D) imaging^8,18^, but this approach has the physical limitations of fluorescent imaging (that is, autofluorescence, a subcellular diffraction limit and fluorophore spectral crowding).

Optical imaging can be achieved not only with visible light but also with shorter wavelength (0.01–10 nm) X-rays, which can penetrate samples and probe tissues nondestructively down to approximately 10-nm resolution. This makes X-rays ideal for repeat imaging of bulk or sectioned biological samples across a range of spatial resolutions (for example, whole human lung down to cellular level 3D imaging^19^). X-ray microscopy can be combined with spectroscopic methods such as element-sensitive X-ray fluorescence (XRF)^20,21^, exploiting the fact that elements with different atomic numbers fluoresce with signature wavelength emissions when excited by an X-ray beam. We reasoned that multiscale, nondestructive X-ray microscopy could achieve fluorescence-type imaging if X-ray-sensitive reporter tags based on different elements, which we call Z-tag reporters, were used. Previous work has demonstrated XRF detection of single molecular markers labeled with antibodies conjugated to gold nanoparticles^22^ and to cadmium-containing quantum dots^23^, but multiplexed X-ray microscopy, as far as we are aware, had not been achieved.

Here, we demonstrate multiplexed, multiscale tissue imaging with X-rays, a strategy we call multielement Z-tag XRF (MEZ-XRF). We repurposed chelating polymers used to conjugate isotope tags to affinity reagents for mass spectrometry-based imaging^24^ as XRF-detectable Z-tags. MEZ-XRF allows detection of high-Z elements at subparts-per-million levels, enabling detection of Z-tag-conjugated primary antibodies with a spectral resolution sufficient to measure multiple neighboring element emissions in parallel. We used MEZ-XRF to image 20 different Z-tags in parallel and in a nondestructive manner in cell line models and in tissues, including breast tumor, tonsil and appendix, and showed that rapid low-resolution overviews can be followed by more sensitive, high-resolution scans for repeated, highly multiplexed imaging of tissue at multiple spatial resolutions. We also combined MEZ-XRF with SABER^8^ Z-tag metal amplification adapted for metals^25^ for 13 markers to achieve either ultrafast imaging speeds or detection of low-abundance markers. In sum, MEZ-XRF enables highly multiplexed imaging with mass tags across multiple biological scales without tissue destruction. This will enable new avenues for multiomic tissue analyses in health and disease.

## Results

### K-edge XRF parallel imaging of high atomic number elements

**Fig. 1:**
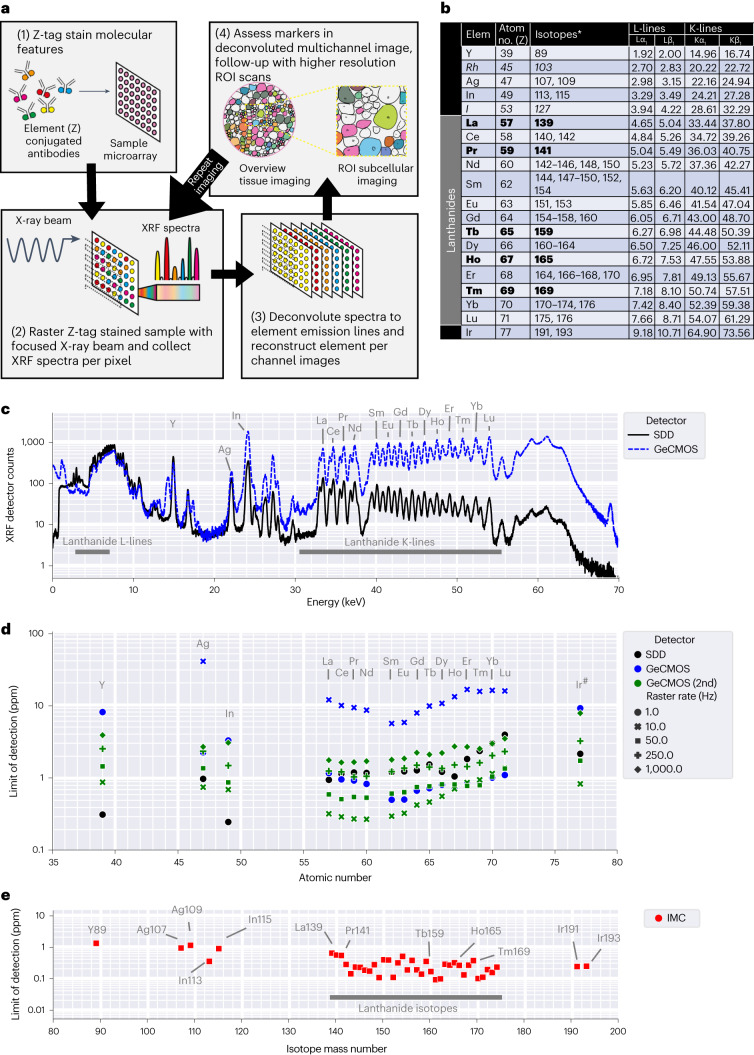
MEZ-XRF principle and element detection limits. **a**, MEZ-XRF involves (1) staining of biological samples with Z-tagged affinity reagents, (2) raster scanning of a focused X-ray beam over the stained sample and collecting emission spectra for each pixel, (3) deconvoluting spectra into multichannel images and (4) analyzing images. Resolution is determined by the focus and step size of the raster X-ray beam. Repeat imaging at different resolutions enables multiscale MEZ-XRF. **b**, The highest yield L- and K-line emissions for the 20 elements used as Z-tags and stable isotopes used for isotope-tagged reagents. All elements were included in the multielement gelatin standard except those in italics. Elements indicated in bold have a single isotope enabling direct comparison between XRF and IMC signal. **c**, Averaged 1-s emission spectra (*n* = 20), triggered by a 69-keV X-ray beam raster scanned over a gelatin standard containing 200 ppm of each Z-tag element (except Rh and I). XRF emissions were recorded with an SDD (black) or GeCMOS (blue) detector. The major Kα_1_ emission lines of Z-tag elements are labeled. **d**, XRF detection limits determined from an eight-point serial dilution series for each element in the multielement gelatin standard as measured by deconvoluted Kα_1_ emissions (^#^Lα_1_ for Ir) with the indicated detectors and raster rates. **e**, IMC detection limits determined from eight-point serial dilution gelatin standards with raster scanning of 500 nm^2^ pixels at 200 Hz. IMC signals from an identifiable isotope of a single element are labeled. Unlabeled lanthanide isotopes are mixtures of isotopes (**b**) that are isotope abundance-adjusted according to their presence in the single-element standard solutions. IMC isotope abundances per element are shown in Extended Data Fig. 3.

To perform MEZ-XRF imaging, FFPE sections of tissue were stained with a Z-tag-labeled antibody panel. Metals were then imaged by raster XRF with a 500-nm diameter X-ray beam and a step size of either 0.5 or 4 µm for a high-resolution scan or an overview, respectively. Element deconvolution yielded 2D multichannel images where each channel corresponds to an emission line arising from a single Z-tagged antibody (Fig. 1a).

**Extended Data Fig. 1:**
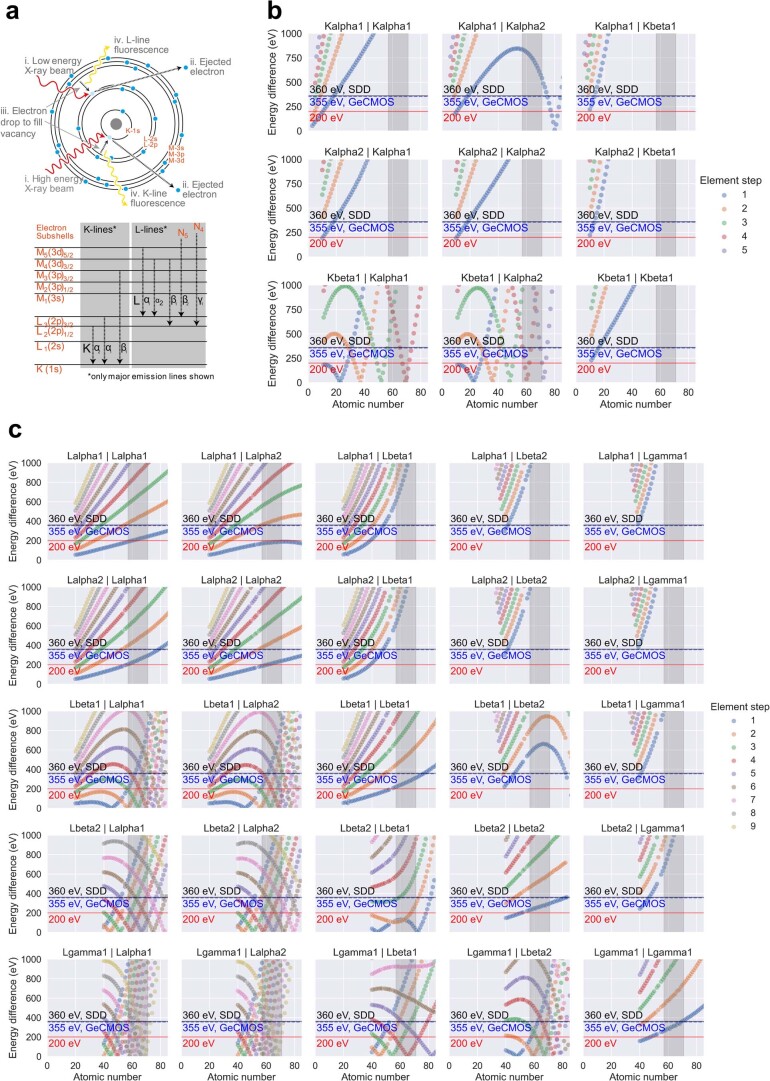
Overlap of X-ray fluorescent emissions. **a, Upper:** X-ray (red) displacement of a core shell electron creates a vacancy; high energy X-rays are able to displace electrons from central shells. If the vacancy is filled by an electron from a surrounding shell, the replacement electron can lose energy as a photon emission (yellow). K-shell fluorescence is generally brighter than L-shell fluorescence as non-fluorescent core shell stabilisation phenomena such as Auger electron loss are less prevalent for innermost shells. An element has multiple emissions (for example, Kα_1_, Lα_1_) that depend on the shell that loses the electron and the shell that supplies the replacement electron. **Lower:** Emission lines that arise from different subshells. **b and c**, The energy gap between the major **b)** K-line or **c)** L-line emissions for an element (element 1) and a neighboring element (element 2) at multiple Z-number element steps (indicated by color above element 1). Subplots are the emission line of element 1 against which the energy difference for an emission line of element 2 is calculated. The black horizontal line at 360 eV and blue horizontal line at 355 eV are the Vortex SDD and Mirion GeCMOS detector full-width half-maximum spectral resolutions, respectively, calculated for the La Kα1 emission line (calibration spectra are shown in Extended Data Fig. 2). The red horizontal line at 200 eV is a best-case spectral resolution for current energy dispersive detectors. The dark grey vertical bar indicates the lanthanides. Note that there are fewer K-line emission overlaps than L-line emission overlaps beneath the spectral resolution limit of each detector in the lanthanide region. Emission line energies from the X-ray data booklet^43^.

XRF identifies different atomic elements based on the specific wavelength of their XRF emissions. Elemental XRF emissions are triggered when an incident X-ray beam displaces a core shell electron to create a core shell vacancy, which, when filled by an outer shell electron, can cause the replacement electron to lose energy that is emitted as an element specific XRF emission (Extended Data Fig. 1a). Multiplexed XRF imaging of element-based Z-tags requires a setup capable of detecting low concentrations of the lanthanide elements on which most Z-tags are based^24^. The method must also distinguish neighboring lanthanide emissions. Different XRF strategies could be used to detect Z-tags as elements have multiple emission lines (that is, low-energy L-lines, high-energy K-lines) depending on the core shell electron initially displaced by the excitatory X-ray beam (Extended Data Fig. 1a). We chose a high-energy, K-shell XRF imaging strategy rather than the more common L-shell XRF approach as few lanthanide K-lines overlap below the spectral resolution limits of the current generation of fast energy-dispersive detectors, whereas there are multiple L-line overlaps that complicate their deconvolution (Extended Data Fig. 1b,c). Well-separated K-shell emissions allow unambiguous identification of multiple Z-tag elements in parallel.

**Extended Data Fig. 2:**
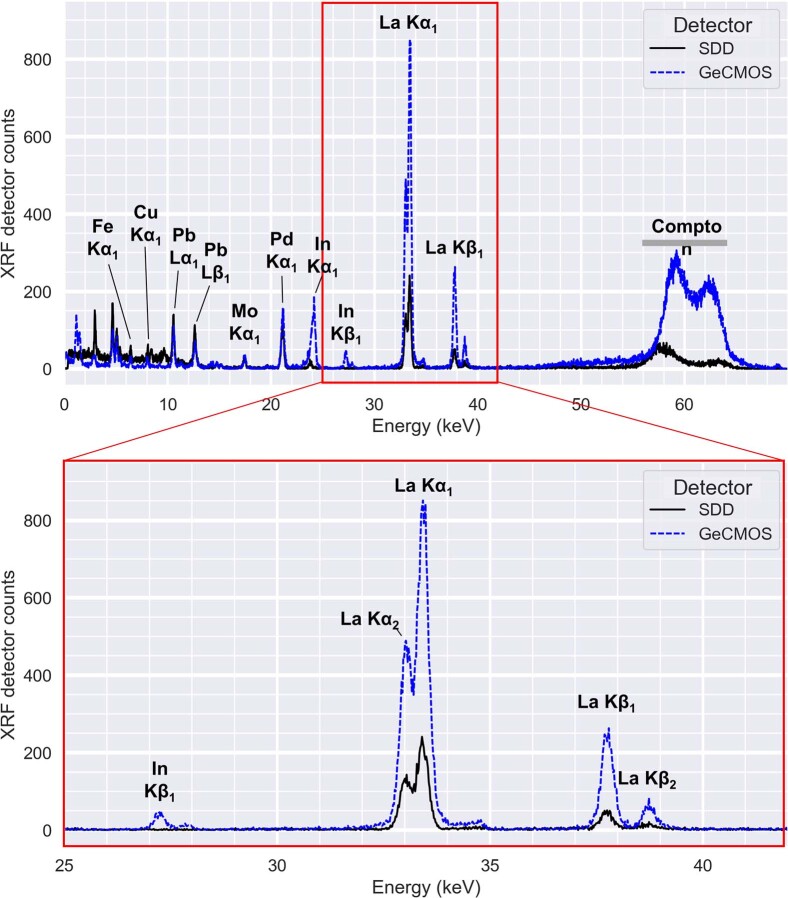
Calibration spectra for SDD and GeCMOS detector. **Upper:** Emission spectra collected on a 1-second exposure of the AXO thin film standard containing Pb, La, Pd, Mo, Cu, Fe, and Si. **Lower:** Zoomed in X-axis region from upper panel. In emissions are from instrument fittings. Spectral resolution for each detector was calculated as the full width at half maximum of the La Kα_1_ peak.

**Extended Data Fig. 3:**
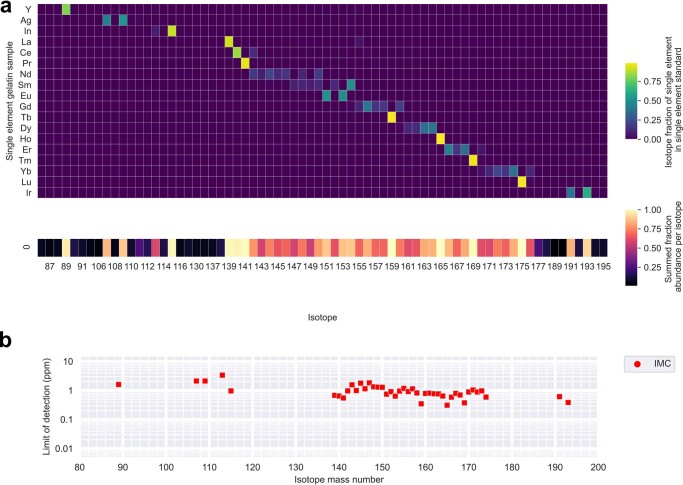
IMC isotope abundance per element. **a, Upper:** Heatmap of the fraction of isotopes in the individual element standards assembled into the multielement gelatin standard used to determine MEZ-XRF and IMC limits of detection as shown in Fig. 1. Each row is the indicated element measured in a single-element gelatin standard at 200 ppm, raster ablated in a 20 x 20-pixel region at 200 Hz with 0.5-µm step. Average IMC counts per gelatin standard were divided by blank gelatin counts, then the fraction of each isotope (per element) was calculated and plotted. **Lower:** Heatmap of the summed abundance fractions for each isotope used to normalize the isotope IMC limits of detection presented in Fig. 1e. **b**, IMC detection limits not corrected for isotope abundance.

To define MEZ-XRF detection limits, we generated an eight-point standard dilution series of lanthanides and other elements suitable for use as Z-tags in gelatin. The concentrations used simulated the range of element concentrations expected in a tissue stained with metal-conjugated antibodies for IMC. The dilution series was imaged with a 69 keV X-ray beam that efficiently triggered lanthanide K-line emissions while separating the highest energy K-line of interest (Lu Kα_1_-line, 54.07 keV) from broad Compton scatter (56–64 keV), which might otherwise mask the highest energy K-line emissions (Fig. 1b). Our initial experiments used a silicon drift detector (SDD), but for later experiments a prototype (GeCMOS) and then a state-of-the-art commercialized germanium (GeCMOS2) detector became available. Germanium detectors have improved high-energy detection limits, due to better high-energy detection efficiency (improved quantum yields) relative to silicon. The SDD and the GeCMOS detectors had full-width at half-maximum spectral resolutions of 360 and 355 eV, respectively, on the La Kα_1_ emission line (33.442 keV) (Extended Data Fig. 2). This was sufficient to spectrally separate those elements within the gelatin standard (Fig. 1c). For the lanthanides, GeCMOS2 offered superior sensitivity over the SDD and enabled detection of 16 elements at subparts-per-million levels at a 10-Hz raster rate (Fig. 1d). In a direct comparison of XRF and IMC on the same multielement gelatin standards, the subparts-per-million lanthanide detection limits of our K-shell XRF approach were similar to those of IMC at 200 Hz when adjusted for isotope abundance per element (Fig. 1e and Extended Data Fig. 3). Thus, our XRF setup has sensitivity comparable to that of IMC.

### MEZ-XRF imaging using Z-tagged antibodies

**Fig. 2:**
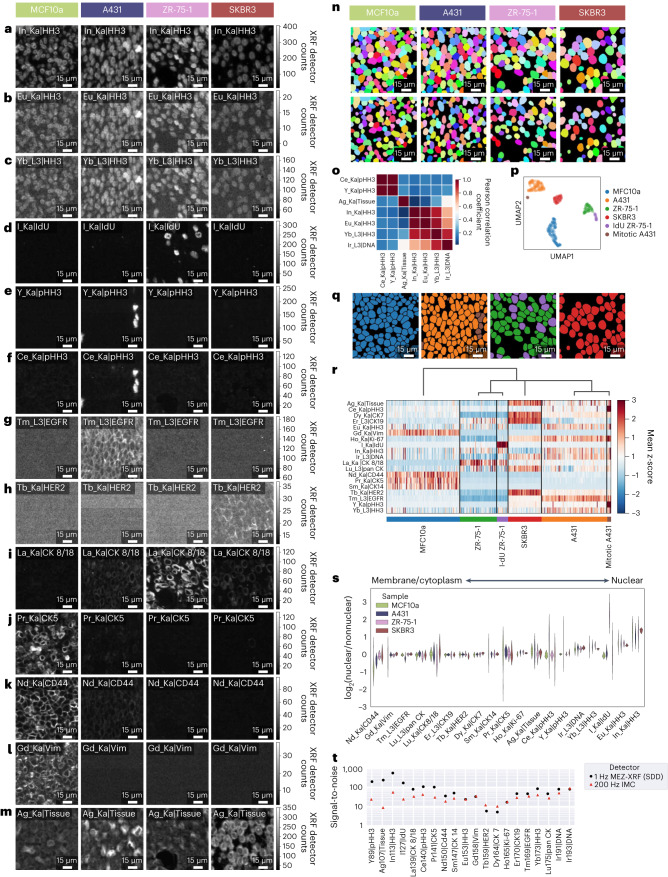
Multiplexed imaging and cytometry of Z-tag-labeled breast epithelial cell lines. **a**–**m**, MEZ-XRF imaging of the markers indicated by element and emission line and antigen symbol (rows) in four breast epithelial cell lines (columns): In_Ka|HH3 (**a**); Eu_Ka|HH3 (**b**); Yb_L3|HH3 (**c**); I_Ka|IdU (**d**); Y_Ka|pHH3 (**e**); Ce_Ka|pHH3 (**f**); Tm_L3|EGFR (**g**); Tb_Ka|HER2 (**h**); La_Ka|CK8/18 (**i**); Pr_Ka|CK5 (**j**); Nd_Ka|CD44 (**k**); Gd_Ka|Vim (**l**) and Ag_Ka|Tissue (**m**). Each column shows the same field of view imaged with a 500 nm focused 69 keV X-ray beam with 500 nm raster steps at 1 Hz. XRF emissions were recorded with an SDD. **n**, Cell (upper panels) and nuclear (lower panels) segmentation masks generated with Mesmer for the four epithelial cell lines. **o**, Pearson correlation heatmap of single-cell intensities of the indicated markers. Labels indicate element emission lines and marker. **p**, Leiden clustering on single-cell intensities of an informative marker (one per cell type), plus Ce-pHH3 and I-IdU. **q**, Cell masks colored by Leiden clusters identified in **p**. **r**, Heatmap of single-cell expression patterns ordered according to the Leiden clusters identified in **p**. **s**, Relative nuclear to nonnuclear intensities for the indicated markers. Markers above axis bars are nuclear, below axis bars are nonnuclear. Violin plots (three dashed horizontal lines per violin show the lower quartile, median value and upper quartile values) show distribution of nuclear/nonnuclear marker ratios for all cells of a single field of view for one sample per cell type. **t**, Signal-to-noise comparison between MEZ-XRF with SDD. The data for the same samples imaged by IMC are shown in Extended Data Fig. 4.

**Extended Data Fig. 4:**
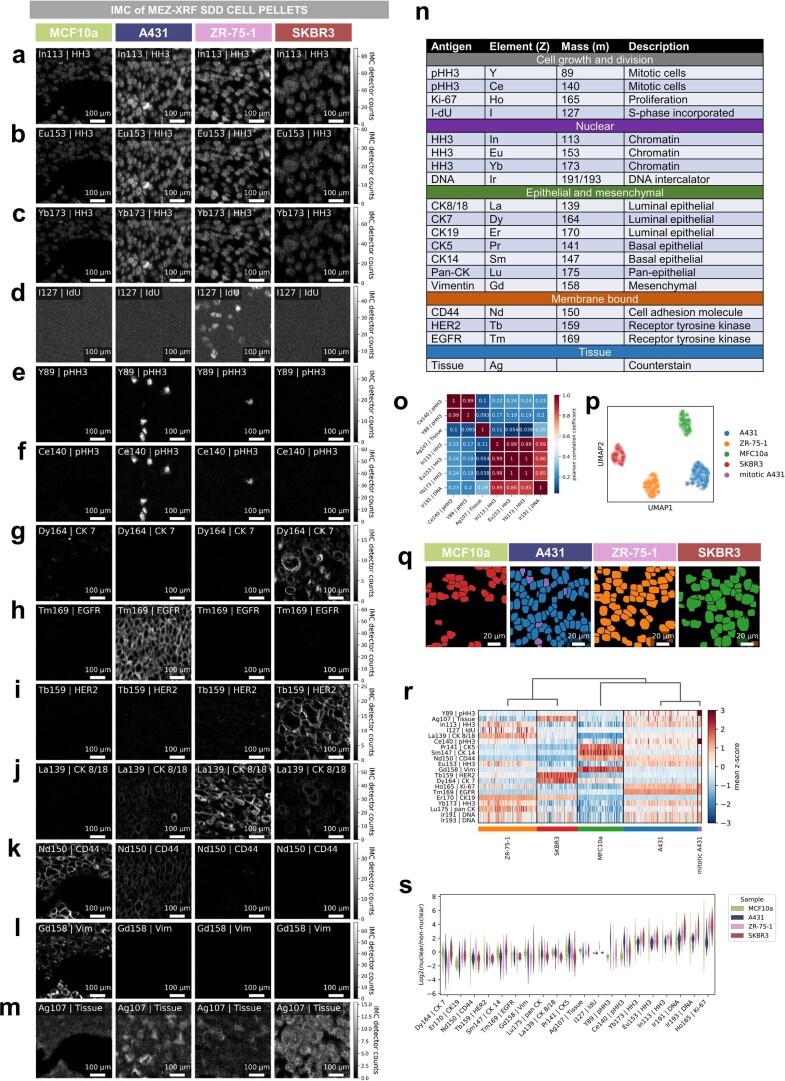
IMC of markers detected in cell pellet samples by MEZ-XRF. **a**, Antigens and element tags used as Z-tags for imaging of breast epithelial cell lines. **b-n**, IMC images of the indicated markers in MCF10a, A431, ZR-75-1, and SKBR3 cell pellets (columns). Samples are presented in columns and element/marker channels in rows. Image labels (for example, In113|HH3) indicate the *isotope* (for example, In113) for the indicated *marker* (for example, HH3). Underlined markers indicate that the same antibody was conjugated to different elements. **o**, Pearson correlation heatmap of the indicated marker intensities for single cells. Labels indicate element isotope and marker. **p**, Leiden clustering on single-cell intensities of an informative marker (1 per cell type) and Ce-pHH3 and I-IdU. **q**, Cell masks coloured by Leiden clusters identified in panel p. **r**, Heatmap of single-cell expression patterns ordered according to the Leiden clusters identified in panel p. **s**, Relative nuclear to non-nuclear intensities derived from nuclear and whole cell segmentations for the top 10% of cells per cell line for each marker. Markers above axis bars are nuclear, below axis bars are non-nuclear. Violin plots (3 dashed horizontal lines per violin show the lower quartile, median value, and upper quartile values) show distribution of nuclear/non-nuclear marker ratios for all cells of a single field of view for 1 sample per cell type.

To assess whether MEZ-XRF enabled imaging of molecular markers labeled with different Z elements, we used Z-tagged antibodies to stain cells from four FFPE epithelial cell lines: mammary MCF10a, breast tumor-derived ZR-75-1 and SKBR3, and the skin tumor-derived A431 (Fig. 2a–m). We subsequently imaged an unmatched region from the same sample using IMC (Extended Data Fig. 4a–m). These cell lines have distinct marker expression profiles including HER2 enrichment in SKBR3, epidermal growth factor receptor (EGFR) enrichment in A431 (ref. ^26^), CK8/18 enrichment in ZR-75-1 (ref. ^27^) and CK5 enrichment in MCF10a cells^28^. Additionally, we manipulated cell lines to establish differential marker expression, collecting A431 cells during the exponential growth phase to enrich them for pHH3^+^ mitotic cells and labeling nuclei of ZR-75-1 cells with the thymidine analog 5-iodo-2′-deoxyuridine (IdU). The antibody panel we used to image these cell line samples included antibodies to these markers and to other common markers such as HH3 (Extended Data Fig. 4n). The panel also included several markers targeted by the same antibody but conjugated to different elements to evaluate how Z-tag sensitivity varied across elements and to orthogonally evaluate the precision of Z-tag marker targeting.

We subjected 4-µm-thick FFPE sections to antigen retrieval, overnight labeling with an antibody panel and nuclear staining with an Ir DNA intercalator followed by an Ag tissue counterstain. Analyses of detector counts from the anti-HH3 antibodies conjugated to three different Z-tags (In, Eu and Yb) showed that lower Z elements provided greater signal-to-noise than higher Z elements (Fig. 2a–c), recapitulating element sensitivity patterns observed using the SDD (Fig. 1d). The similar staining patterns detected for the three anti-HH3 antibodies tagged with the different elements confirmed that Z-tag labeling did not interfere with specificity of the antibody.

All Z-tags had the expected marker differences between cell lines: iodine-positive nuclei were only identified in IdU-pulsed ZR-75-1 cells (Fig. 2d), and mitotic cells were detected only in proliferating A431 cells (Fig. 2e,f). The EGFR signal was highest in A431 cells (Fig. 2g), and we only detected HER2 in SKBR3 cells (Fig. 2h). The broad differences in tissue origins of these cell lines were reflected in variable expression of CK8/18 (that is, high in ZR-75-1, low in SKBR3 and A431, absent in MCF10a cells) and in enrichment of certain markers in nontumorigenic MCF10a cells (CD44, vimentin and CK5) but not in the tumorigenic cell lines A431, ZR-75-1 and SKBR3 (Fig. 2i–l). As expected, EGFR, HER2, CK8/18, CK5, CD44 and vimentin localized to cell membranes and cytoplasm (Fig. 2g–l), Ag counterstain was visible throughout cells (Fig. 2m) and HH3 was detected in nuclei (Fig. 2a–c). Subsequent IMC of unmatched regions of the same samples showed the same patterns (Extended Data Fig. 4a–m). These results confirmed that MEZ-XRF recapitulated the expected biological distributions of evaluated markers.

We reasoned that MEZ-XRF could be used for quantitative profiling of marker intensities in the cell line models. We segmented individual whole cells and nuclei using cytoplasm and/or membrane and nuclear marker channels with the deep learning-based cell segmentation model Mesmer^29^, and then constructed matched cell and nuclear masks for single-cell comparisons and assessment of Z-tag subcellular localization (Fig. 2n). The intensities of the three HH3-targeting Z-tagged antibodies were highly correlated with each other and with the nucleus-targeting Ir-based DNA intercalator at the single-cell level, and duplicate pHH3 channels were also mutually correlated (Fig. 2o). An observed correlation between Ag and Ir signals was due to a technical artifact, as Ag precipitated in the presence of Ir resulting in colocalized flakes of Ir and Ag; we therefore did not use the Ag counterstain in subsequent experiments. Subsequent IMC of the same samples demonstrated the same correlation patterns as MEZ-XRF (Extended Data Fig. 4o).

Leiden clustering^30^ separated the different cell lines as well as a mitotic pHH3^+^ subset of A431 cells and the IdU^+^ subset of ZR-75-1 cells (Fig. 2p,q). Leiden clustering of IMC data of these same markers with the same parameters identified the same cell-type clusters, except for the IdU^+^ ZR-75-1 cluster, possibly due to lower signal intensity for ^127^I in the IMC experiment (Extended Data Fig. 4p–r). The phenotypic separation of the nontumorigenic MCF10a cells from the three tumorigenic cell lines was also visible for both data types (Fig. 2r and Extended Data Fig. 4r). Per marker, for the top 10% of expressing cells across the four cell types, known nuclear and cytoplasm and/or membrane markers were enriched in the expected subcellular regions for both MEZ-XRF and IMC data (Fig. 2s and Extended Data Fig. 4s). Finally, we compared signal-to-noise between MEZ-XRF and IMC for each marker by measuring the difference between the mean signal of the top 10% of cells and the mean signal of the lowest 10% of cells across the four cell types (Fig. 2t). There was comparable cellular signal between the two methods, with superior signal-to-noise for 1 Hz MEZ-XRF using the SDD for the lower mass channels. Together, these data demonstrate that MEZ-XRF can be used for cytometry and subcellular localization analyses of multiple markers.

### MEZ-XRF enables multiplexed nondestructive tissue imaging

**Fig. 3:**
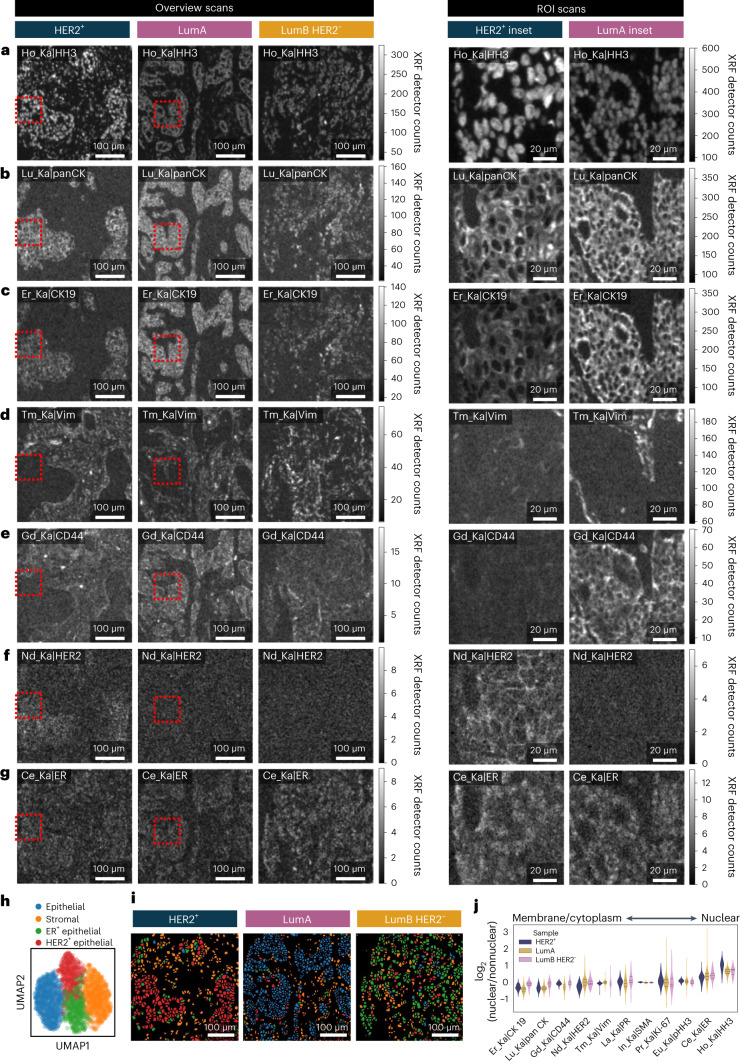
MEZ-XRF enables molecular marker imaging of human FFPE tissue. **a**–**g**, MEZ-XRF images of the indicated markers (rows) in HER2^+^, luminal A (LumA) and luminal B HER2^−^ (LumB HER2^−^) tissues (columns): Ho_Ka|HH3 (**a**); Lu_Ka|panCK (**b**); Er_Ka|CK19 (**c**); Tm_Ka|Vim (**d**); Gd_Ka|CD44 (**e**); Nd_Ka|HER2 (**f**) and Ce_Ka|ER (**g**). Overview scans were imaged with a 500 nm focused 69 keV X-ray beam with 2 µm raster steps at 20 Hz. ROI (indicated by red box and shown magnified to the right) were imaged with a 500 nm focused 69 keV X-ray beam with 0.5 µm raster steps at 5 Hz. XRF emissions were recorded with a prototype GeCMOS detector. Images are 0.5–99.5% gray levels of detector counts per element emission line matched across samples. **h**, Leiden clustering of single cells segmented from overview images colored by clusters as annotated by marker enrichment. **i**, Leiden cluster annotations (colors as in **i**) projected back onto single-cell segmentation masks of representative overview imagery for the indicated samples. **j**, Subcellular localization of molecular markers in each tumor sample. The relative nuclear to nonnuclear intensities were derived from nuclear and whole-cell segmentations of ROI scans. Violin plots (three dashed horizontal lines per violin show the lower quartile, median value and upper quartile values) show distribution of nuclear/nonnuclear marker ratios for all cells of a single field of view for one sample per diagnosis.

To test MEZ-XRF on physiologically relevant samples, we imaged biopsy core punches from a HER2^+^ tumor, a luminal A tumor and a luminal B HER2^−^ tumor after staining for key breast cancer diagnostic markers as well as for epithelial, stromal and immune markers (Fig. 3a). Taking advantage of the nondestructive capabilities of MEZ-XRF, we first rapidly imaged entire cores (20 Hz, 2-µm steps) to provide overview scans and then performed higher sensitivity ROI scans (5 Hz, 0.5-µm steps).

**Extended Data Fig. 5:**
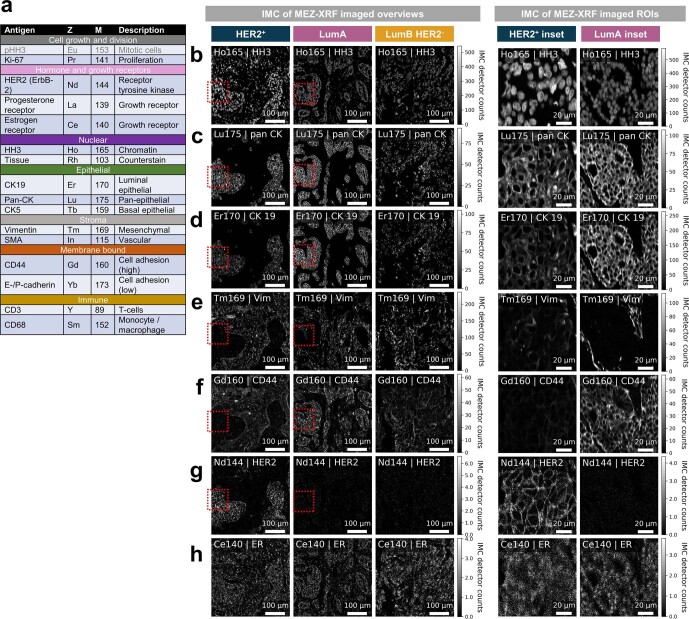
IMC of markers detected in breast cancer samples by MEZ-XRF. **a**, The antibody panel used to label the indicated molecular features in breast cancer samples from Fig. 4 with additional tag isotope information. **b-h**, IMC images of the indicated markers in breast cancer samples. Samples are presented in columns and element/marker channels in rows. The fields of view are the same as those imaged in Fig. 3 by MEZ-XRF, here images obtained subsequently by IMC are presented. Samples were scanned once with a 500-nm step (pixel size) raster at 200 Hz. Overviews and ROIs were manually selected from complete scans to match the regions shown in Fig. 3. Overview scans were down sampled 1:4 to match the 2-µm step sampling used for MEZ-XRF overviews. Images are 0.5–99.5% grey levels of IMC counts per isotope matched across samples as shown in colour bars.

**Extended Data Fig. 6:**
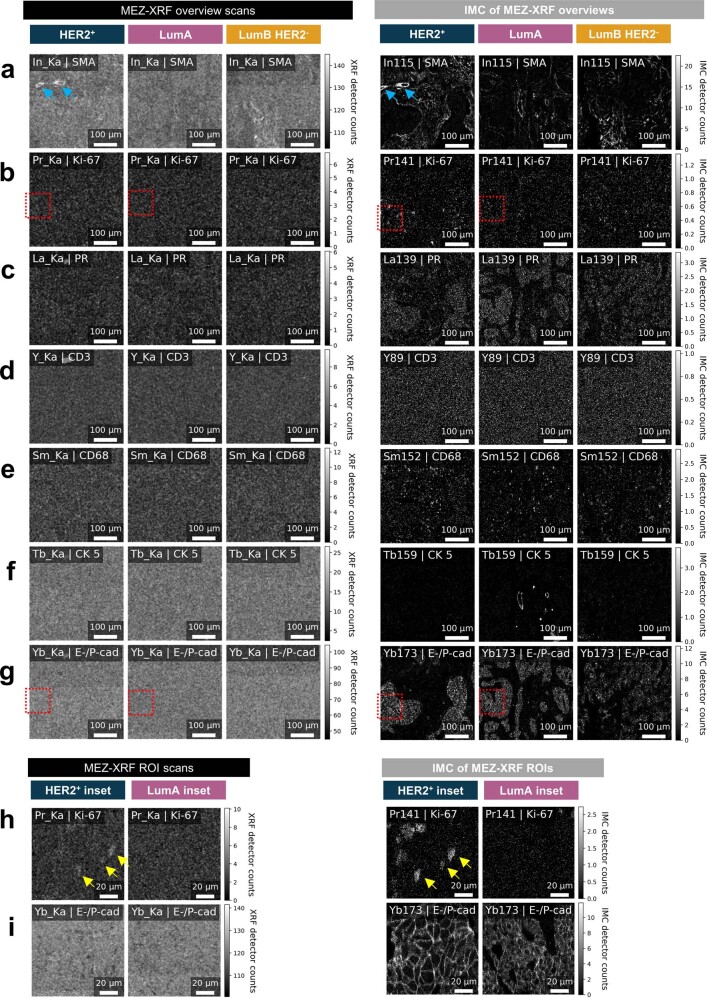
MEZ-XRF and matched IMC images of additional markers in breast cancer samples. **a-j**, Matched MEZ-XRF (left) and IMC (right) overviews (top) and ROIs (bottom). Samples are presented in columns and element/marker channels in rows. MEZ-XRF labels indicate *element_emission lines/marker* (as in Fig. 2a–m). IMC labels indicate *isotope/marker* (as in Extended Data Fig. 5). Blue arrows indicate SMA^+^ vasculature, yellow arrows indicate PR^+^ cells identified in both MEZ-XRF and IMC images. MEZ-XRF overviews were imaged with a 500-nm diameter spot focused X-ray beam with 2-µm raster steps at 20 Hz. MEZ-XRF ROIs were imaged with a 500-nm diameter spot focused X-ray beam with 500-nm raster step at 5 Hz. MEZ-XRF images are 0.5—99.5% grey levels of detector counts per element emission line matched across samples as shown in colour bars. IMC images were scanned once after MEZ-XRF using a 500-nm step (pixel size) with raster ablation at 200 Hz. Overview scans were down sampled 1:4 to match the 2-µm step sampling used for MEZ-XRF overviews. IMC images are 0.5–99.5% grey levels of IMC counts per isotope matched across samples as shown in colour bars.

Highly expressed molecular markers such as nuclear HH3, epithelial panCK, luminal epithelial CK19 and stromal vimentin as well as low-abundance markers such as CD44 were detected in overview scans (Fig. 3b–f). Expression of these proteins was confirmed in higher sensitivity scans that also revealed membrane localization of CD44 (Fig. 3f). HER2 was detected at low levels in epithelial cells of the HER2^+^ sample but not in the luminal A or luminal B HER2^−^ sample, as expected (Fig. 3g). Higher sensitivity scans of the HER2^+^ region in the HER2^+^ sample confirmed that HER2 expression was localized to cell membranes (Fig. 3g). The ER protein was detected at low levels in all three samples, with more intense staining in the luminal B HER2^−^ sample than in other samples (Fig. 3h). Subsequent imaging of the same samples by IMC revealed the same intensity patterns and subcellular localization as observed in MEZ-XRF images (Extended Data Fig. 5). For markers with relatively low expression as measured by MEZ-XRF, subsequent IMC of the same samples confirmed marker absence or very low signal (Extended Data Fig. 6a–j). With the exceptions of the lowest abundance markers that were only be detected by IMC (Extended Data Fig. 6h–i), cell phenotypes were consistent between MEZ-XRF and IMC.

To evaluate the suitability of MEZ-XRF for tissue phenotyping, we quantified the frequencies of identified cellular phenotypes. Cells were segmented in images from overview scans with Mesmer^29^, using the HH3 nuclear channel and an aggregated cytoplasm and/or membrane marker channel (panCK, CK19, fibronectin and Rh tissue counterstain), followed by Leiden clustering^30^. We observed HER2^+^, HER2^+^/ER^−^ and ER^+^ epithelial clusters in the HER2^+^, luminal A and luminal B HER2^−^ samples, respectively (Fig. 3i), as expected, and these clusters were located in the epithelial regions of each sample (Fig. 3j). Stromal cells were identified in all three samples (Fig. 3i), showing the lack of batch effects between scans. Finally, based on cells segmented from ROI scanned at higher sensitivity, we confirmed that MEZ-XRF quantifiably distinguished cellular distributions of detectable molecular markers. As expected, HH3, pHH3, ER and Ki-67 were located in nuclear regions; CK19, panCK, CD44 and HER2 were located in or near the membrane, and vimentin and SMA were detected in both nuclear and membrane regions, likely reflecting a cytoplasmic localization (Fig. 3k). In summary, our data demonstrate that MEZ-XRF can be used to nondestructively and repetitively image tens of markers in parallel in tissue at multiple scales and can quantitatively discriminate different tissue phenotypes.

### Ultrafast MEZ-XRF imaging with SABER-amplified Z-tags

**Fig. 4:**
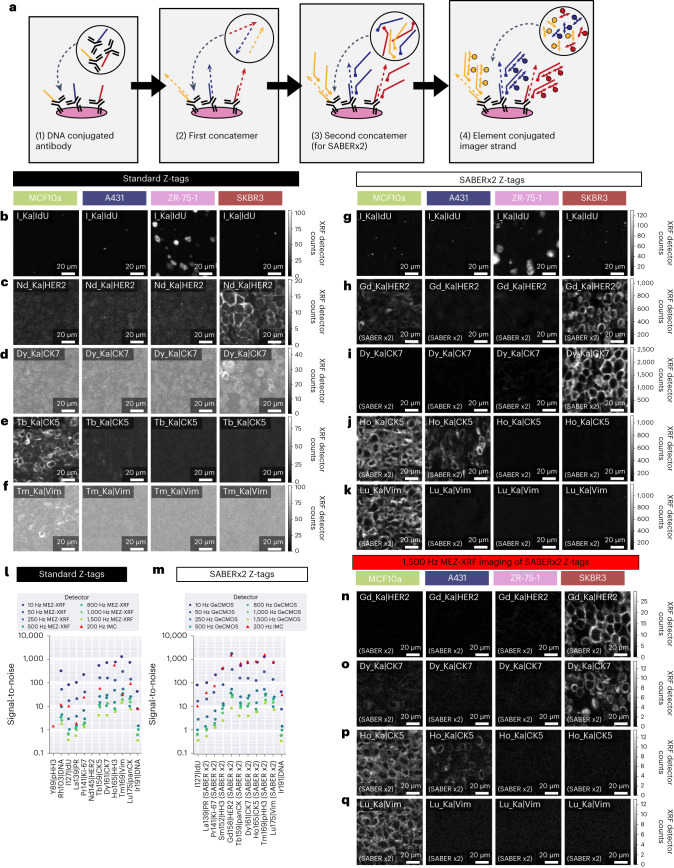
SABER Z-tags increase speed and sensitivity of MEZ-XRF. **a**, Schematic of SABER Z-tag element amplification. **b**–**f**, Standard MEZ-XRF imaging of the indicated markers in MCF10a, A431, ZR-75-1 and SKBR3 cell pellets (columns): I_Ka|IdU (**b**); Nd_Ka|HER2 (**c**); Dy_Ka|CK7 (**d**); Tb_Ka|CK5 (**e**) and Tm_Ka|Vim (**f**). Scans were imaged with a 500 nm focused 69 keV X-ray beam with 500 nm raster steps at 10 Hz. **g**–**k**, SABERx2-amplified MEZ-XRF imaging of the indicated markers in MCF10a, A431, ZR-75-1 and SKBR3 cell pellets: I_Ka|IdU (**g**); Gd_Ka|HER2 SABERx2 (**h**); Dy_Ka|CK7 SABERx2 (**i**); Ho_Ka|CK5 SABERx2 (**j**) and Lu_Ka|Vim SABERx2 (**k**). Scans were imaged with a 500 nm focused 69 keV X-ray beam with 500 nm raster steps at 10 Hz. **l**, Signal-to-noise comparison between standard MEZ-XRF and IMC. IMC images are shown in Extended Data Fig. 7a. **m**, Signal-to-noise comparison between SABERx2 MEZ-XRF and IMC. IMC images are shown in Extended Data Fig. 7b. **n**–**q**, High-speed (1,500 Hz) SABERx2-amplified MEZ-XRF imaging of the indicated markers in MCF10a, A431, ZR-75-1 and SKBR3 cell pellets: Gd_Ka|HER2 (**n**); Dy_Ka|CK7 SABERx2 (**o**); Ho_Ka|CK5 SABERx2 (**p**) and Lu_Ka|Vim SABERx2 (**q**).

We next sought to improve both detection of markers and imaging speed of MEZ-XRF. We used two approaches. First, we increased the element concentration targeted to each marker by signal amplification with SABER^8,25^. This method was selected for its ability to amplify multiple antibody markers in parallel. In SABER, each antibody is conjugated to an oligonucleotide, which then binds complementary single-stranded DNA concatemers (SABERx1) with optional use of a second complementary strand to further amplify the signal (SABERx2). The SABERx2 approach achieves more amplification than a single amplification round^25^, and we used it for further experiments. The complementary strands have multiple binding sites for Z-tagged imager strands, enabling exponential amplification of metal label levels per marker (Fig. 4a). Second, we used the state-of-the-art GeCMOS2, which has a tenfold better detection limit than the prototype GeCMOS (Fig. 1d). The use of the GeCMOS2 detector improved signal intensities at 10 Hz relative to slower 1 Hz measurements using the SDD (compare Fig. 4b,c to Fig. 2d,i).

**Extended Data Fig. 7:**
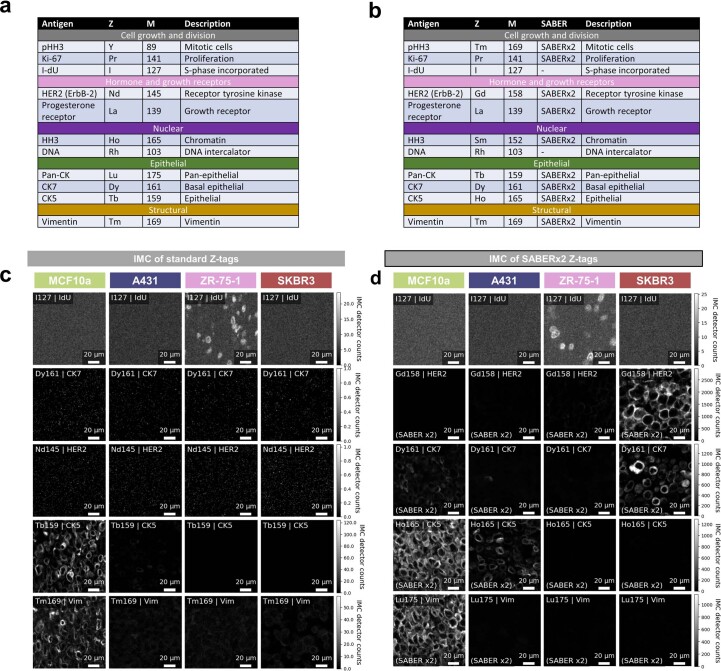
IMC images of standard and SABERx2 Z-tags in cell pellets. **a**, Standard conjugation panel. **b**, SABERx2 amplified conjugation panel. **c and d**, Matched IMC imaging of four epithelial cell lines (columns) labelled with **c)** standard Z-tags or **d)** SABERx2 amplified Z-tags. Top left label indicates *isotope/marker* (as in Extended Data Fig. 5). Bottom left indicates whether SABERx2 was used. IMC images were scanned once after MEZ-XRF using a 500-nm step (pixel size) with raster ablation at 200 Hz. These are the same sections as imaged by MEZ-XRF in Fig. 4.

To assess the effects of SABER Z-tag amplification on MEZ-XRF sensitivity, serial sections from the epithelial cell pellets were stained with antibodies conjugated to standard Z-tags (Fig. 4c–f and Extended Data Fig. 7a) or were stained with antibodies conjugated to oligonucleotides detected using the SABERx2 protocol (Fig. 4h–k and Extended Data Fig. 7b). Incorporated IdU levels were not amplified, hence show comparable signal levels between standard and SABERx2 imaged samples (Fig. 4b,g). For comparison, these samples were also imaged by IMC (Extended Data Fig. 7c,d). Using antibodies conjugated to standard Z-tags, markers detected using 10-Hz MEZ-XRF such as HER2 and CK7 yielded no signal by IMC (compare Fig. 4c,d and Extended Data Fig. 7c). Further, 10-Hz MEZ-XRF with GeCMOS2 showed superior signal-to-noise to IMC (Fig. 4l) meaning that MEZ-XRF with GeCMOS2 achieved signal-to-noise equivalent to standard 200-Hz IMC. Use of SABERx2 increased signal-to-noise relative to the antibodies conjugated to standard Z-tags for both MEZ-XRF and IMC (Fig. 4m and Extended Data Fig. 7). Thus, SABERx2 conferred higher sensitivity to both techniques for markers with otherwise borderline detection such as CK7 (Fig. 4d,i and Extended Data Fig. 7c,d).

**Extended Data Fig. 8:**
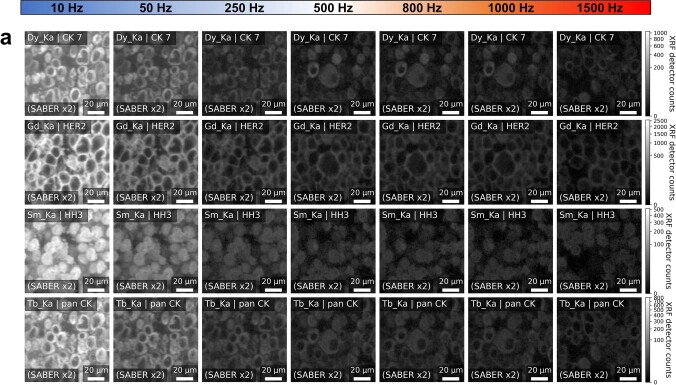
MEZ-XRF imaging of SABER Z-tags in cell pellets imaged at different speeds. **a-d**, MEZ-XRF images of SKBR3 cells at the imaging rate indicated above each column. Colour scale is cubed root. Scans were imaged with a 500-nm focused 69-keV X-ray beam with 500 nm raster steps at the indicated speed. Top left label indicates *element_emission lines/marker* (as in Fig. 2a–m). Bottom left indicates that SABERx2 was used.

Given the increased signal-to-noise ratios of SABERx2-amplified MEZ-XRF, we stepwise increased imaging speed to assess the speed limits of our system. Marker signal declined proportionally to dwell time (Fig. 4l–m), but nevertheless markers were still clearly detectable at 1.5 kHz (Fig. 4n–q and Extended Data Fig. 8). With the setup available, we could not increase raster speed further due to software latency limitations of reading full spectra from the GeCMOS2 detector.

**Fig. 5:**
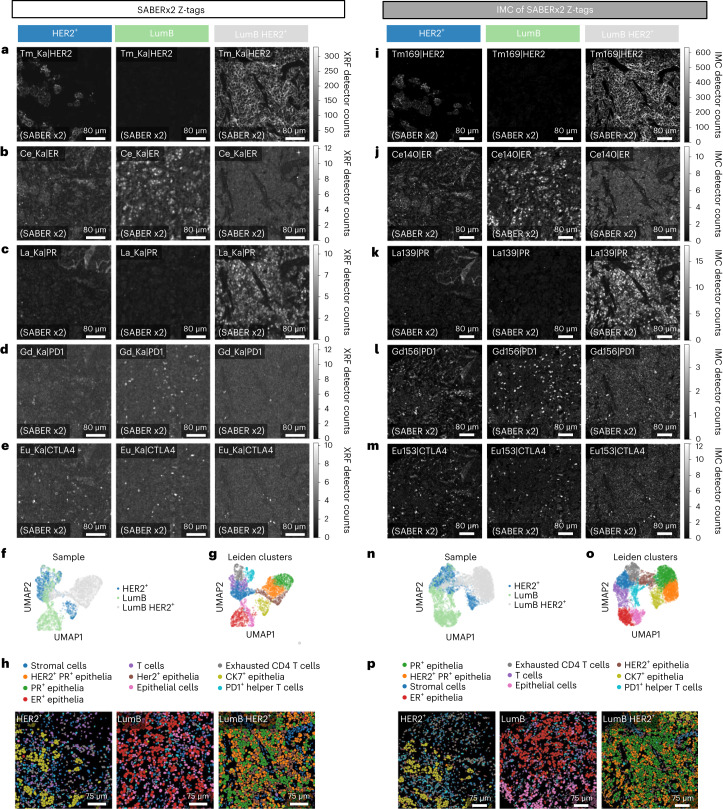
MEZ-XRF with SABERx2 Z-tags enables imaging of low-abundance markers in breast tumor tissue. **a**–**e**, MEZ-XRF imaging of SABERx2 Z-tags in breast tumor tissue in HER2^+^, luminal B (Lum B) and luminal B HER2^+^ (LumB HER2^+^) samples (columns): Tm_Ka|HER2 (**a**); Ce_Ka|ER (**b**); La_Ka|PR (**c**); Gd_Ka|PD1 (**d**) and Eu_Ka|CTLA4 (**e**). Scans were imaged with a 500 nm focused 69 keV X-ray beam with 500 nm raster steps at 50 Hz. For all panels, each row is a particular marker. The top left indicates the marker and element in the Z-tag. The bottom left indicates SABERx2 amplification level. All XRF emissions were recorded with the state-of-the-art GeCMOS2 detector. **f**,**g**, UMAP of MEZ-XRF samples colored by sample (**f**) or by Leiden cluster annotations of different cell types (**g**). **h**, Leiden clusters projected back onto single-cell segmentation masks for each sample with colors as in **g**. **i**–**m**, IMC imaging of the same sections in regions overlapping those imaged in **a**–**e**: Tm_Ka|HER2 (**i**); Ce_Ka|ER (**j**); La_Ka|PR (**k**); Gd_Ka|PD1 (**l**) and Eu_Ka|CTLA4 (**m**). For all panels, each row is a particular marker. Top left indicates the marker and element in the Z-tag. Bottom left indicates SABERx2 amplification level. **n**,**o**, UMAP of IMC samples colored by sample (**n**) or by Leiden cluster annotations (**o**) of different cell types. **p**, Leiden clusters projected back onto single-cell segmentation masks for IMC imaged samples with colors as in **n**.

**Extended Data Fig. 9:**
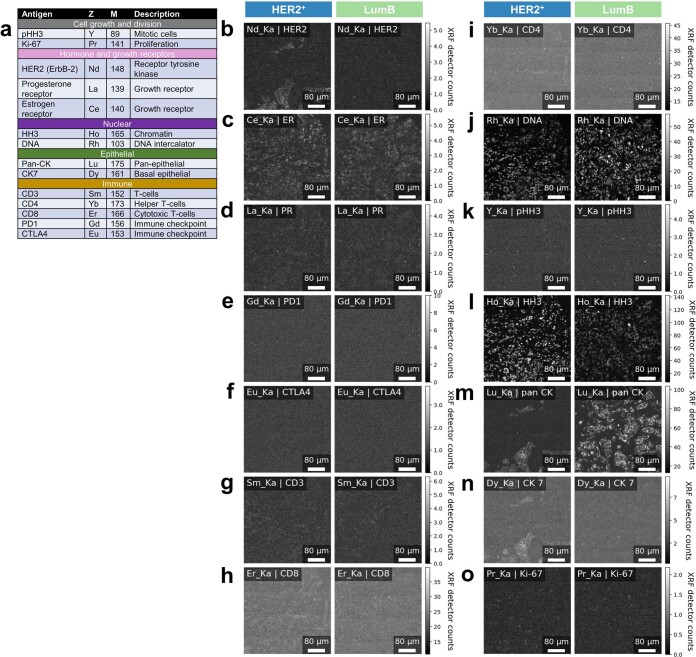
MEZ-XRF imaging of standard Z-tags on breast tumor tissue. **a**, Antibody panel used for standard Z-tags. **b-o**, MEZ-XRF images of breast tumour tissues stained with standard Z tags. Columns indicate the different tissue samples, and rows indicate markers and element/emission lines. All XRF emissions were recorded with state-of-the-art GeCMOS2. Scans were imaged with a 500-nm focused 69-keV X-ray beam with 500-nm raster steps at 50 Hz. Top left label indicate *element_emission lines/marker* (as in Fig. 2).

**Extended Data Fig. 10:**
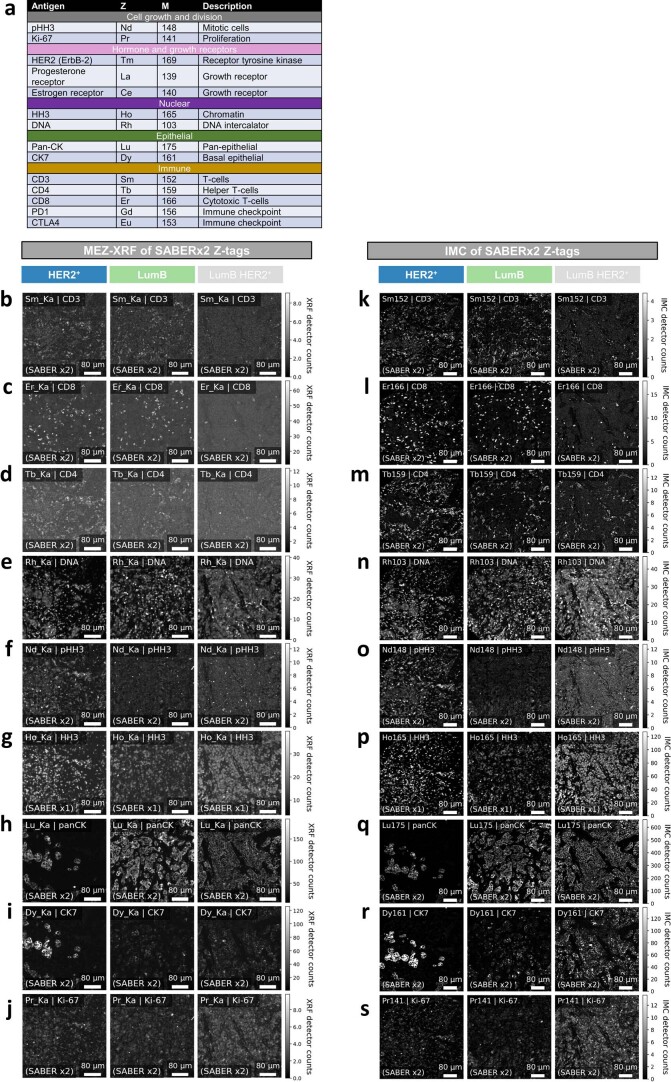
MEZ-XRF imaging of SABERx2 Z-tags in breast tumor tissue. **a**, Antibody panel used for SABER Z-tags. **b-j**, MEZ-XRF imaging of breast tumour tissues stained with SABER-Z tags. Columns indicate the different tissue samples, and rows indicate markers and element/emission lines. All XRF emissions were recorded with state-of-the-art GeCMOS2. Scans were imaged with a 500-nm focused 69-keV X-ray beam with 500-nm raster steps at 50 Hz. Top left label indicate *element_emission lines/marker* (as in Fig. 2). Bottom left indicates whether SABERx1 or SABERx2 was used (a decision based on the expected marker amplification level given our experience with these reagents). **k-s**, IMC of the sections imaged by MEZ-XRF. Top left label indicates *isotope/marker*. Bottom left indicates whether SABERx1 or SABERx2 was used (a decision based on the expected marker amplification level given our experience with these reagents).

In addition to dramatically improving imaging speed, SABERx2 combined with GeCMOS2 made detection of low-abundance molecular targets possible. In breast cancer tissue samples, several markers that were not detectable with standard Z-tags (Extended Data Fig. 9) were detectable with SABERx2 Z-tags (Fig. 5a–e). These markers included PR (Fig. 5c and Extended Data Fig. 9d), the immune regulators PD1 (Fig. 5d and Extended Data Fig. 9e) and CTLA4 (Fig. 5n and Extended Data Fig. 9f), and the T cell markers CD3, CD4 and CD8 (Extended Data Fig. 10b–d and Extended Data Fig. 9g–i). Single-cell analysis of these data distinguished ten cell clusters including multiple epithelial cell types (ER^+^, PR^+^, HER2^+^) consistent with the original diagnosis (Fig. 5f–h). In addition, T cell subtypes, including CD3^+^CD4^+^CTLA4^+^PD1^+^ T cells (likely exhausted regulatory T cells) and CD3^+^CD8^+^PD1^+^ exhausted cytotoxic T cells, were identified in the immune-infiltrated HER2^+^ and luminal B samples (Fig. 5f–h). Subsequent IMC of an overlapping region from the same samples demonstrated similar staining intensities to those in the MEZ-XRF data (Fig. 5i–m and Extended Data Fig. 10k–s). The analyzed regions did not completely overlap, and there were image resolution and channel sensitivity differences between IMC and MEZ-XRF that led to cell segmentation differences that complicated a one-to-one cell comparison between methods. However, we observed consistencies between methods, notably that the same cell phenotypes were identified and that distributions of cell phenotypes across sample types was the equivalent in images from both methods (Fig. 5g,h,o,p). Overall, our data support the use of MEZ-XRF as a cytometric tool.

### Multiscale correlative imaging by MEZ-XRF

**Fig. 6:**
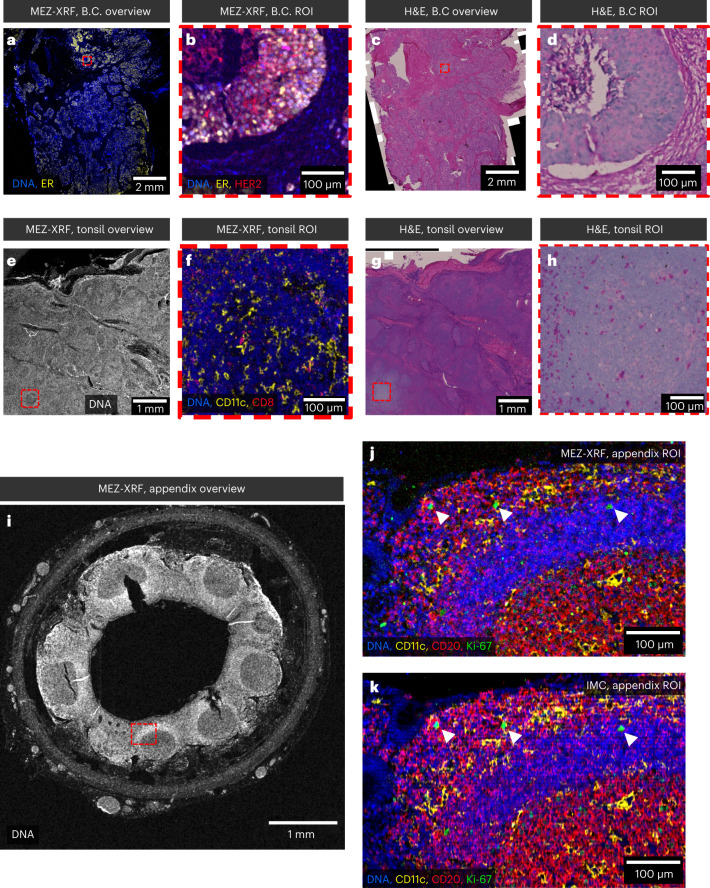
Multiscale correlative imaging by MEZ-XRF. **a**, SABERx2 MEZ-XRF overview of a 1 cm^2^ region of breast cancer tissue. **b**, SABERx2 MEZ-XRF image of ROI of outlined in red in **a**. **c**, Image of section shown in **a** that was H&E stained after MEZ-XRF. **d**, Image of region outlined in red in **c**. **e**, SABERx2 MEZ-XRF overview of a 1.1 cm^2^ region of tonsil tissue. **f**, SABERx2 MEZ-XRF image of tonsil follicle in region outlined in red in **e**. Scale bar, 100 μm. **g**, Image of section shown in **e** that was H&E stained after MEZ-XRF. **h**, Image of region outlined in red in **g**. **i**, SABERx2 MEZ-XRF overview of appendix. **j**, MEZ-XRF image of region outlined in red in **i**. **k**, IMC of the region outlined in red in **i** performed after MEZ-XRF. The image was filtered with a Gaussian column-shaped filter to reduce row artifacts introduced during IMC ablation. White arrowheads highlight Ki-67-positive cells.

Tissue biopsies are often available on the cm scale, but the limited throughput of multiplexed platforms means that only limited ROI from these sections are evaluated. Selection of ROI is usually guided by hematoxylin and eosin (H&E) or low-plex immunofluorescent staining of an adjacent section. The nondestructive multiscale nature of MEZ-XRF means that low-resolution overviews can be used to guide selection of ROI based on multiplexed molecular information in combination with tissue structure. Different combinations of scan speeds and step sizes can be used to image large areas at low sensitivity, and ROI within the overview can be imaged at high sensitivity (example scan speeds in Supplementary Table 1). To demonstrate the multiscale capabilities of MEZ-XRF, we performed an overview scan of 1 cm^2^ breast cancer section at 200 Hz with 4-µm step size, which took about 8.7 hours (Fig. 6a). We then imaged an ER-rich, 400 µm^2^ ROI at 5 Hz with 0.5-µm step size, which took about 8.8 hours (Fig. 6b). The high-sensitivity scan detected additional markers including HER2 (Fig. 6b). After MEZ-XRF, the sample remained suitable for H&E staining and the MEZ-XRF images were aligned based on the H&E-stained image (Fig. 6c,d). H&E images revealed bleaching of the eosin stain in the ROI-imaged area, but nuclei could be identified.

A similar approach was used to generate an overview of tonsil (5 mm^2^, 100 Hz, 4-µm step size, roughly 4.3 hours). A CD11c-rich follicle was identified and scanned at higher sensitivity (Fig. 6e,f), and the sample was stained with H&E to allow alignment (Fig. 6g,h). We also imaged a complete transverse section of appendix by MEZ-XRF (5 mm^2^, 100 Hz, 4-µm step size, roughly 4.3 hours, Fig. 6i), followed by high-sensitivity imaging of a CD20-rich region (240 µm^2^, 25 Hz, 0.5-µm step size, roughly 10.7 hours, Fig. 6j). This region was subsequently evaluated by IMC (Fig. 6k). After filtering the IMC image with a column-shaped Gaussian filter as described in detail in Methods section to correct for parallel raster artifacts (potentially due to autofocus issues on the Mylar film), the high-resolution MEZ-XRF image and the IMC image had comparable signal localization and signal-to-noise levels. These data highlight the general applicability of MEZ-XRF for imaging of different immunolabeled tissues and demonstrate that nondestructive MEZ-XRF can be combined with additional imaging modalities.

## Discussion

MEZ-XRF is a next-generation bioimaging method that enables highly multiplexed, targeted molecular contrast for X-ray bioimaging. The method is a new approach to tagged molecular microscopy that enables nondestructive, high-speed imaging spanning the tissue- to subcellular-resolution range. Although previously described light or nonoptical imaging approaches achieve imaging in the tissue-to-cellular or cellular-to-subcellular ranges, MEZ-XRF is a nondestructive, highly multiplexed platform that images across tissue-to-subcellular scales with the capacity for ultrafast kHz imaging when combined with SABER Z-tag reagents. Since fluorescent emissions of Z-tags are measured with a tissue-penetrant X-rays, MEZ-XRF can be further developed for high-resolution or 3D imaging, as it is free from the spectral crowding and diffraction limits of visible light fluorescence microscopy.

Such multiscale, multiplexed imaging within a single platform makes new experiments possible. For instance, MEZ-XRF could be used to study signaling and physical interactions between neighboring rare cell types, which must first be localized in a larger tissue. More generally, overview scans could guide ROI selection to efficiently capture phenotypes of interest^31^. Indeed, its nondestructive nature means that MEZ-XRF images could be combined with other modalities such as H&E staining (as we demonstrated) or spatial transcriptomics to reveal tissue structure, guide cell segmentation or enable transfer learning between the same tissue section from small multiplex ROI to whole tissue. MEZ-XRF could also be used to combine the multiplexing advantages of mass tags with versatile fluorescent imaging strategies. For instance, barcode-based or iterative staining of Z-tags could be used to further expand MEZ-XRF multiplex capacity and would simply require an approach to liberate metal tags after imaging such as UV-mediated or chemical cleavage of metal-reagent linkers.

Z-tag atomic fluorescence offers key advantages over fluorophore tags. Discrete element emissions are readily multiplexed and, unlike photolabile fluorophores, atomic fluorescence does not degrade, making repeated imaging rounds possible. As MEZ-XRF is based on atomic-level fluorescence, once sensitivity issues are solved, miniaturized Z-tags (for example, metal nanoparticles) will also make it possible for MEZ-XRF to achieve molecular resolution. Capitalizing on this high-resolution potential will be possible with short-wavelength X-ray beams that can already be focused to less than 20 nm in diameter^21,32^. The main hurdle to reducing resolution at this stage is the need for fabrication of reflective high-energy X-ray beam optics that maintain the high flux necessary for subparts-per-million metal sensitivity.

MEZ-XRF detection limits differ per element and per detector. Our characterization of MEZ-XRF detection limits with SDD and germanium detectors will inform future marker panel design, as highly expressed markers should be placed in the channels with the weakest signal-to-noise. A dual detector approach with SDD and GeCMOS approaches could achieve high sensitivity across the energy range of Z-tags. More elements could be incorporated to increase multiplexing capacity or could be combined with imaging of endogenous elements to study sample physiology with tagged marker context.

At 10 Hz, the GeCMOS2 detector has element and isotope sensitivity better than 200 Hz IMC, making it possible to detect high-abundance targets (for example, vimentin in breast fibroblasts, estimated at 2,900.7 normalized transcripts per million (nTPM)^33^). A potential issue for the germanium detectors is that escape peaks are more intense than with the SDD, which could introduce signal interference^34^. However, this did not appear to be an issue in our analysis, possibly due to the low concentrations of element per Z-tag used in our experiments. Moreover, escape peaks for a germanium detector are accounted for in our PyMCA spectral deconvolution configuration^35^. We showed that SABERx2 amplification of metal signals^25^ increased sensitivity 10–100-fold relative to standard Z-tags, enabling detection of low-abundance epitopes (for example, T cell CTLA4 at 41.9 nTPM and PD1 34.7 at nTPM^33^).

For both MEZ-XRF and IMC, SABERx2 conferred improved marker sensitivity, which for MEZ-XRF, enabled faster imaging. Only in the case of anti-HH3 (D1H2 clone) was SABERx2 intensity lower than the unamplified standard antibody, perhaps because this clone yields one of the brightest unamplified signals. In practice, we recommend checking the level of staining achieved by unamplified, SABERx1 amplified, and SABERx2-amplified methods to choose the best signal-to-noise approach per marker, particularly for MEZ-XRF where signal intensity and signal-to-noise affect the practical imaging speed.

Using SABERx2, we achieved marker-specific imaging up to 1.5 kHz. This is better than comparable mass spectrometric technologies. For IMC, the maximum reported rate is 400 Hz (ref. ^36^); with raster speed limited by aerosol movement from raster ablated samples to the detector, an issue that MEZ-XRF does not have. For MIBI, the maximum reported raster rate is 83 Hz (four rounds of 3 ms per pixel, totaling 12 ms per pixel not accounting for raster dwell time^37^), and the rate of secondary ion generation requires multiple passes of an imaged region to achieve desired sensitivity.

Further MEZ-XRF speed gains could be achieved with a higher flux excitatory X-ray beam, which could be achieved with wider bandwidth, multilayer monochromators for a tenfold flux boost. Sensitivity could also improve at the detector level by using compound or larger solid angle detectors that capture more emissions^38,39^. Speed could also be gained by scanning with multiple beams in parallel^40^.

Despite comparable signal-to-noise levels between MEZ-XRF and IMC, in some instances background levels appear higher in MEZ-XRF images. This is due to a combination of factors including how each technology handles detector counts. The IMC detector has a count intensity threshold, below which a pixel intensity is set to zero (histograms for IMC images are counts above zero), producing a uniform dark background. However, for MEZ-XRF, there is a bell curve of pixel intensities around the average of the background. An additional background contributor for MEZ-XRF in the higher Z channels is Compton scatter, which raises overall counts.

The low-energy deposition of the high-energy, 69 keV X-ray beam we used to image samples with low Z elements meant we could use very high flux density X-ray beams to trigger lanthanide XRF emissions with negligible damage to our biological samples. The K-shell approach also simplified our setup, as samples were imaged in air under atmospheric conditions, simplifying sample changes. Indeed, a high-energy, K-line strategy is also well suited to 3D approaches due to the excellent sample penetrance of high-energy X-rays. 3D MEZ-XRF would require a method for 3D labeling of samples with metal-conjugated reagents and, to be practical, would need faster imaging to collect the multiangle images needed for tomographic reconstruction.

Although high-energy, K-line XRF imaging is largely restricted to specialized high-energy beamlines at synchrotron facilities, accessible XRF options exist including miniature, high-energy X-ray sources^41,42^. Alternatively, lanthanide L-line emission imaging with high spectral resolution fluorescent detectors could make possible Z-tag L-line imaging with existing commercial laboratory sources such as the AttoMap (Sigray) or with electron microscopes albeit with lower sensitivity than XRF^1^. For instance, wavelength dispersive detectors (roughly 10 eV resolution versus more than 200 eV resolution for energy-dispersive detectors), ultra-high-resolution calorimetric detectors, or a recently demonstrated hybrid wavelength dispersive approach demonstrated to have approximately 12-eV spectral resolution at 4.5 keV (ref. ^43^) would be ideal for L-line lanthanide-based Z-tag imaging.

In summary, the MEZ-XRF system, optimized for detection of lanthanide-based Z-tags in biological samples, is a nondestructive, highly multiplexed approach for imaging of element-tagged biological molecules in cells and tissues. Technological advances in X-ray sources, optics, detectors and element-tag reagents promise to deliver further speed and sensitivity gains and to make MEZ-XRF more broadly accessible. The uniquely scalable resolution and depth-imaging properties of X-rays, the stability and miniaturization potential of Z-tags, and the high speeds possible with MEZ-XRF make possible high-speed, highly multiplexed imaging across tissue, cells and molecular resolutions.

## Methods

### Gelatin standards

XRF and IMC detection limits were measured using a twofold, eight-point dilution series of gelatin-embedded elements^44^. Briefly, a 50 mg l^−1^ master mix of select elements was prepared in 2% nitric acid (v/v, doubly distilled H_2_O) from the following certified, single-element (1,000 mg l^−1^) concentration standards: Y (Sigma, catalog no. 01357), Ag (Sigma, catalog no. 12818), In (Merck, catalog no. 1703240100), La (Sigma, catalog no. 11523), Ce (Sigma, catalog no. 16734), Pr (Sigma, catalog no. 59947), Nd (Sigma, catalog no. 04730), Sm (Merck, catalog no. 1703480100), Eu (Sigma, catalog no. 05779), Gd (Sigma, catalog no. 05660), Tb (Sigma, catalog no. 44881), Dy (VWR, catalog no. 68339), Ho (Sigma, catalog no. 01541), Er (Sigma, catalog no. 05693), Tm (Sigma, catalog no. 01496), Yb (Sigma, catalog no. 39956), Lu (Sigma, catalog no. 03909) and Ir (VWR, catalog no. 455502R). The dilutions of the element master mix were made in 2% nitric acid. A freshly prepared 10% (m/v) solution of 300-bloom gelatin (Sigma, catalog no. 2500) dissolved at 55 °C in ultrapure MilliQ water was mixed with each element dilution (or nitric acid for blank) in a two to one ratio of gelatin to element. Aliquots of 5 µl of each mix were spotted onto Mylar film (Chemplex, catalog no. 3016) or onto glass slides to create the 200, 100, 50, 25, 12.5, 6.25, 3.125, 1.5625 and 0 ppm dilution series. Mylar films and glass slides spotted with these standards were covered with a glass Petri dish and dried in a 100 °C convection oven for 1 h.

### Cell culture and microarray preparation

Breast epithelial cell lines ZR-75-1 (CRL-1500), MCF10a (CRL-10317), SKBR3 (HTB-30) and A431 (CRL-1555) were sourced from the American Type Culture Collection. Cell lines were cultured in 150-mm dishes in media and conditions recommended by the American Type Culture Collection. Cell lines were propagated to roughly 80 million cells before collection at roughly 80% confluency, except for the A431 cell line, which was collected at roughly 40% confluency to enrich for mitotic cells. At 24 h before collection, the ZR-75-1 cells transiting S-phase were pulse labeled with 20 µM of IdU (Sigma, catalog no. I7125) by adding 400 µl of a 1 mM IdU stock to the 20 ml of culture.

To prepare cell pellets, cell lines were separated from culture dishes by incubation with 5 ml of TrypLE (Thermo Fisher, catalog no. 12604). After 5–15 min, depending on the cell line, TrypLE was deactivated by addition of 10 ml of PBS. Cell suspensions were spun down at 250*g* for 5 min and resuspended in 5 ml of PBS. Cells were counted and transferred to 5-ml Eppendorf tubes. Cell suspensions were spun down at 250*g* for 5 min, supernatants were removed by aspiration and cells were resuspended in 83.75 µl of plasma (Sigma, catalog no. P9523) per 50 million cells. Next, 145 µl of thrombin (catalog no. T4648-1KU) per 50 million cells was added. After incubation at room temperature for 10 min, cell pellet clots were formed.

To prepare FFPE samples, cell pellet clots were transferred to a mesh grid CellSafe Biopsy Insert (VWR, catalog no. 100501-266), placed in an embedding cassette and submerged in 10% formalin (EMS, catalog no. 15710) overnight. After a 1-h wash in doubly distilled H_2_O, embedding cassettes were transferred to 15% sucrose. After 1 h at room temperature, cassettes were transferred to 30% sucrose and kept at 4 °C overnight. Cassettes were stored in 70% ethanol for no longer than 2 days before automated embedding on a spin tissue processor (Epredia, Thermo Fisher). In the spin processor, cell pellets in embedding cassettes were dehydrated through a graded ethanol series (ethanol:deionized water, 70:30, 80:20, 90:10, 96:4, 96:4 and 100:0; 1 h each), xylene infiltrated through three 2-h incubations and paraffin embedded by three 2-h incubations in 64 °C liquid paraffin. Cell pellets were cast to paraffin blocks, and 1-mm diameter cores were punched from solid paraffin blocks and assembled into a T-Sue cast microarray (EMS, catalog no. 69132-01).

### Ethical approval

A breast cancer tissue microarray was assembled from 500-nm diameter core punches of anonymized FFPE biopsies taken at the University Hospital Zurich; clinical data were associated with each sample. FFPE blocks for breast tumor, tonsil and appendix were prepared at University Hospital Zurich. These were provided under ethics approval no. KEK-ZH-Nr 2014-0425.

### Element-tag immunolabeling

Sections of 4 µm thickness cut from the cell pellet FFPE microarray and the breast cancer microarray were collected on either glass slides or 6-µm thick Mylar film. The film was spread taut by pressing between an inner and outer 3D nylon support made by selective laser sintering (designs available in Supplementary Data 1). Tissue sections collected on Mylar films or glass slides were dried onto respective surfaces on a heat bed at 37 °C for 24 h.

For immunostaining, tissue sections were dewaxed by washing three times for 10 min in Histo-Clear (EMS, catalog no. 64110-01), then rehydrated by three 5-min 100% ethanol washes followed by washes with a graded ethanol series (ethanol:deionized water, 96:4, 96:4, 90:10, 90:10, 80:20 and 70:30; 3 min each), followed by transfer to Tris-buffered saline (TBS), pH 7.6. Heat-induced epitope retrieval was conducted in Tris-EDTA, pH 9 in a Decloaking Chamber (Biocare Medical, catalog no. DC-2012) heated to 95 °C for 20 min. After antigen retrieval, samples were left to cool for 20 min at room temperature, before being transferred to room temperature TBS for 20 min.

For immunostaining, tissue sections on the Mylar films or glass slides were circled with a PAP pen (a hydrophobic barrier pen). After blocking with 3% bovine serum albumin (BSA) in TBS for 1 h, samples were stained overnight at 4 °C with either the cell pellet or the breast cancer (Supplementary Tables 2–4) primary antibody panel. Antibody solutions were prepared in TBS, 0.1% Triton X-100 and 1% BSA. Antibodies were conjugated to elements using the Maxpar X8 metal chelating polymer kit (Fluidigm, catalog no. 201300) as per the manufacturer’s instructions. After overnight incubation, samples were washed twice with TBS and optionally stained for 10 min with a nuclei staining Ir DNA intercalator (Fluidigm, catalog no. 201192A) or Rh DNA intercalator (Fluidigm). Sections were washed once with TBS for 5 min and in some cases counterstained with 10 µM AgNO_3_ (Sigma, catalog no. 209139) in doubly distilled H_2_O for 5 min. This silver stain was only used in initial experiments, as we found that Ag precipitated in the presence of Ir resulting in colocalized flakes of Ir and Ag, confounding its use as a counterstain. Sections were then dipped in doubly distilled H_2_O to remove salt crystals and dried overnight at room temperature. For XRF, samples on Mylar films were carefully cut from the frames and taped to 1-mm thick acrylic windows suitable for XRF imaging.

### SABER-amplified element-tag immunolabeling

SABER was used to amplify element concentrations targeted to markers of interest^25^. The antibody clones, bridge DNA sequence IDs, concatemer sequence IDs, imager sequence IDs and isotope labels used to achieve this are summarized in Supplementary Table 5. DNA sequences for all the barcode IDs are available in Supplementary Table 6. Antibodies in SABER panels were conjugated to a bridge DNA sequence via click reaction (azide-modified antibody and DBCO-modified DNA)^25^. Briefly, antibodies were modified and purified with azide using the Glyclick azide activation kit (Genovis) as per the manufacturer’s protocol. Subsequently, 10 molar equivalents of 5′-DBCO-modified DNA (Microsynth) was added to 1 mg ml^−1^ of azide-modified antibody in PBS solution and kept at room temperature overnight to complete the click reaction. Conjugated antibody was purified with a 50-kDa Amicon Ultra Filter (Millipore, catalog no. UFC510096).

For SABER immunostaining, tissue sections were dewaxed and subjected to heat-induced antigen retrieval. Sections were blocked with 3% BSA in PBS for 1 h, then stained overnight at 4 °C with a SABER antibody panel (Supplementary Table 5). SABER panels were prepared in blocking buffer containing 0.2 μg ml^−1^ sheared salmon sperm DNA (Thermo Fisher Scientific, catalog no. AM9680) and 2% dextran sulfate (Sigma, catalog no. D8906). Excess antibodies were removed by three 10-min PBS washes. The remaining antibodies were crosslinked to tissue with 5 mM α,ω-Bis-NHS-PEG (molecular weight of 2,000, Sigma, catalog no. 713783) in PBS at 4 °C for 3 h. Crosslinker was quenched in TBS at room temperature for 20 min.

Next, samples were incubated at 37 °C for 1 h with concatemer solution in a humidified chamber. Concatemers were synthesized as reported^8,25^. Before adding primers, the reaction mixture was prepared to final concentrations of 800 U ml^−1^ of BST LF polymerase (NEB, catalog no. M0275M), 600 μM each of dATP/dTTP/dCTP (NEB, catalog nos. N0441S, N0440S, N0443S), 0.1 to 2 μM of hairpin (IDT), 100 nM of Clean.G hairpin (5′-CCCCGAAAGTGGCCTCGGGCCTTTTGGCCCGAGGCCACTTTCG-3′, IDT) and 10 mM of MgSO_4_ (NEB, catalog no. B1003S) in PBS. The reaction mixture was preincubated at 37 °C for 15 min to remove excess deoxyguanosine triphosphate that could inhibit the reaction. Subsequently, primers were added to a final concentration of 1 μM, and the reaction was kept between 25–37 °C for 2–24 h followed by heat inactivation of polymerase at 80 °C for 20 min (exact conditions for different concatemers are summarized in Supplementary Table 7.

Concatemers were purified and concentrated with a Minelute kit (Qiagen, catalog no. 28004) into 10 μM water. A concatemer solution was prepared in 2× SSC buffer (Sigma, catalog no. S6639) with 25% formamide (Sigma, catalog no. F9037), 10% dextran sulfate, 0.1% (v/v) Tween-20 (Sigma, catalog no. P9416) and 0.2 mg ml^−1^ sheared salmon sperm DNA. Concatemers were typically diluted to 80–100 nM, and concatemers for different targets were mixed and incubated simultaneously. After incubation, samples were washed once with 50% formamide in PBS for 5 min at room temperature and three times with TBS containing 0.1% Triton X-100 at 37 °C for 10 min for each wash.

For SABERx2 amplification, concatemer solution was prepared in the same buffer as used for the first round concatemers and incubated in a humidified chamber at 37 °C for overnight. The second and third concatemers were typically diluted to 100 nM, and concatemers for different targets were mixed and incubated simultaneously. Excess concatemers were removed by washing once with 40% formamide in PBS for 5 min at room temperature and then three times with TBS containing 0.1% Triton X-100 at 32 °C for 10 min each wash.

Finally, samples were incubated with imager solution containing element-conjugated imager strands at room temperature for 1 h in a humidified chamber. Imagers were synthesized as described in the companion paper^25^. Briefly, the MaxPar X8 Antibody labeling kit (Fluidigm) was used to prepare metal-isotope-modified polymer as per the manufacturer′s manual. Chelation was completed by incubating MaxPar X8 polymer in 2.5 mM lanthanide chloride solution (Fluidigm) at 37 °C for 30 min, and the product was purified into C-buffer, provided with the labeling kit using 0.5-ml, 3-kDa Amicon Ultra Filters (Millipore, catalog no. UFC500396). In parallel, 5 nmol of 5′-thiol-modified imager DNA (Microsynth) was activated using 50 mM TCEP at 25 °C for 30 min, ethanol precipitated and resuspended in C-buffer. The activated 5′-thiol-modified imager DNA (1.5–2 nmol) and purified isotope-labeled MaxPar X8 polymer (one tube) were incubated together in 200 μl of C-buffer at 25 °C for 2.5 h and were then purified into 40 μl of water using 0.5-ml Microcon 30 centrifugal filters (Millipore, catalog no. MRCF0R030). The filter-based purification was repeated three times.

Imager solution was prepared in PBS containing 0.1% Triton X-100. Imager probes were typically diluted to 1 μM final concentration. After incubation, samples were washed once with PBS containing 0.1% Triton X-100 for 5 min at 37 °C and twice with TBS for 5 min at room temperature. For nuclear staining, samples were incubated with 10 µM Rh intercalator in TBS for 5–10 min, followed by a 15-min wash in TBS at room temperature. Samples were then dipped into deionized water for a few seconds, dried immediately using pressured air flow and stored at room temperature until measurements. For XRF, samples on Mylar films were carefully cut from the frames and taped to 1-mm thick acrylic windows suitable for XRF imaging.

### XRF imaging

XRF imaging was conducted at beamline ID15A of the European Synchrotron (ESRF). A 69-keV X-ray beam was focused to 500 × 500 nm with Kirkpatrick–Baez mirror optics, delivering roughly 1 × 10^12^ photons s^−1^ at the focal spot. XRF emissions were detected with either dual Vortex (Hitachi) SDDs mounted opposite each other at 90° to the X-ray beam or with a single thermoelectrically cooled Canberra (Mirion Technologies) GeCMOS detector mounted at 90° to the X-ray beam. Prototype and commercialized versions of the GeCMOS detector were used in experiments, with the latter (GeCMOS2) having a longer detector-to-cooling unit probe and improved collimator for shielding that reduced background and improved signal-to-noise relative to the prototype. For each XRF experiment, a thin-film silicon nitride reference standard (AXO, catalog no. RF-200-0510-C00-X) containing Pb, La, Pd, Mo, Cu, Fe and Si was analyzed during 20 lots of 1-s XRF exposures. Detectors were calibrated according to the La Kα_1_ emission line peak, and the spectral resolution of each detector was calculated as the full-width at half-maximum of the La Kα_1_ peak.

For sample mounting, the acrylic windows holding tissue sections or gelatin standards were glued to the top of a glass capillary that was placed on a Huber goniometer on top of the sample stage. For the GeCMOS2 detector, the acrylic windows were directly immobilized to a Huber goniometer. The goniometer was adjusted to align the sample vertically, and the sample was rotated 30–40° relative to the beam path for a clear path for XRF emissions from sample to detector. SDDs were brought to roughly 30 mm distance from the sample. The prototype GeCMOS detector was used at roughly 85 mm due to bulk lead shielding around the germanium probe, meaning the germanium detector (78.5 mm^2^) covered an XRF emission solid angle 3.1 times less than the SSDs (30 mm^2^). We corrected for this difference in presented results by multiplying germanium counts by 3.1. The 2D-XRF raster scans were conducted by collecting emissions while moving the sample stage such that the sample raster stepped through the X-ray focal spot. Emissions collected per step were recorded as multichannel spectra in .hdf file format with scan metadata and additional sensor data from an X-ray diode (‘fpico3’) positioned beside the sample to monitor beam flux (which drops roughly 2% between 2-h synchrotron electron top-up cycles).

During the beamtime, multichannel spectra calibrated with the AXO thin-film standard were deconvoluted to 2D element emission line maps with the PyMCA GUI^35^. These 2D element maps were used to identify ROIs. All scans used the 500 × 500 nm focus beam. Resolution was adjusted by modifying the stage raster step size, and XRF sensitivity was adjusted by modifying the per step dwell time.

### XRF image processing

A Python (v.3.8.8)-based pipeline (key packages listed in Supplementary Table 8)^45–49^ was assembled for processing .hdf file outputs for each scan into 2D element emission lines, which were stacked into a 3D array (*x*, *y*, emission_line). Full Jupyter notebooks^50^ with details of the pipeline and a Conda environment.yml file to run the pipeline are available at https://github.com/BodenmillerGroup/MEZ_XRF. Notebook 1 includes instructions for stitching together scans collected in multiple .hdfs files due to interruption by a beamline-related event. Notebook 2 details the method for deconvolution of per-pixel spectra with PyMCA to 3D image stacks (*x*, *y*, emission_line). Notebook 3 has instructions for normalization of image stacks to beam intensity fluctuations with an fpico_mask generated from the fpico3 diode recordings. The remaining notebooks have the instructions necessary for replication of downstream analysis and reproduction of figures presented in this paper.

Deconvolution was performed with a Python implementation of the PyMCA package with custom configuration files (detector calibration and elements to deconvolute). Detector spectra deconvolution configuration files and raw .hdf spectra are available at Zenodo (10.5281/zenodo.7949102)^51^. For each scan, the deconvoluted raw image stack, a fpico_normalised image stack (to normalize within scan beam intensity fluctuations) and a Compton_scatter_normalised image stack (to facilitate between scan comparisons) were stored as 3D array datasets under their italicized names in the images node of a custom .hdf file with a structure that we term high_plex_hdf that includes images, sample metadata, a Pandas dataframe .hdf export of channel names and a masks node that stores masks (Single-cell and single-nucleus segmentation section) associated with the image stacks. These high_plex_hdf containers keep related images and masks together for each scan, simplifying data sharing and streamlining downstream image processing. These scripts are available in the github repository available with this paper.

### XRF and IMC limits of detection

To define the sensitivities of XRF and IMC, all methods measured 20 × 20 pixels of the eight-point gelatin dilution series with 500-nm raster steps. IMC was obtained by raster ablation at 200 Hz, and XRF raster sampling was performed at 1, 10 or 100 Hz using the dual SDDs or GeCMOS prototype. The same standards were later imaged at higher speeds with GeCMOS2. For all gelatin standard scans, an eight-point calibration curve fit to mean isotope counts (IMC) or mean element emission line counts (XRF) was plotted, and the intercept to blank background^52^ (mean_blank_ + 1.645(s.d._blank_) for the respective channel was used to determine the limit of detection for that channel. For XRF with the SDD or GeCMOS detectors and for IMC, the gelatin blank was used to calculate the limit of detection. For the GeCMOS prototype, the AXO calibration sample was used as the blank (with La and Ce blanks set to that of adjacent Pr due to the presence of La in the AXO sample) as it was the only thin-film blank sample imaged at the different raster rates during that beamtime setup.

### Single-cell and single-nucleus segmentation

For single-cell analysis, XRF image stacks were segmented using the pretrained deep learning segmentation model Mesmer from the Deepcell v.0.9.0 package^29^. A nuclei_mask and a cell_mask were generated for each scan using Mesmer with default parameters and a specified pixel size. Mesmer operates on a two-channel 2D input (a nuclear and a cytoplasmic and/or membrane channel), with one channel or the average of multiple channels mean averaged for these two-channel inputs. The 0.5–99.5% pixel intensity values for each channel were normalized to between 0 and 1, then the average of these normalized channels was used for Mesmer segmentation. Channels that gave good nuclear or cell membrane staining were averaged in this manner for cell segmentation input. Cell pellets were segmented with the HH3 nuclear channel and an averaged CK8/18, CD44, HER2 and EGFR cytoplasm–membrane channel. Breast cancer scans were segmented with a HH3 nuclear channel and an averaged CK19 and panCK cytoplasm–membrane channel.

Mesmer can identify cells lacking nuclei (the nucleus may be out the imaging plane). To evaluate marker distribution in a single cell, cell and nucleus object labels had to be paired. We did this by identifying strongly overlapping cell and nucleus labels in the nuclei_mask and cell_mask (Jaccard score greater than 0.4) to obtain a nuclei_matched_mask and cell_matched_mask in which matching nuclei and cell objects had the same cell ID. A non-nuclei_matched_mask (cell_matched_mask minus nuclei_matched_mask) for each cell ID was also generated. All masks for each scan were stored under their italicized names in the masks node of the high_plex_hdf container.

### Single-cell marker correlation

Single-cell measurements for each cell ID of the cell_matched_mask were made using Steinbock v.0.5.2 (ref. ^53^) using mean intensities per element emission channel from the Compton scatter-normalized images. Measurements were output to an anndata object for single-cell analysis with Scanpy v.1.7.2 (ref. ^54^) with rows (observations) as cell IDs and columns (variables) as mean intensities per channel. Antigens targeted in duplicate or triplicate by different element–antibody conjugations were Pearson correlated from these measurements for each cell ID.

### Single-cell visualization and clustering

In Scanpy, a uniform manifold approximation and projection (UMAP) was used to visualize high-dimension single-cell expression profiles^55^, and Leiden graph clustering was used to identify cell types^30^. For cell pellet and breast cancer datasets, this analysis was applied to a set of informative markers normalized to the 2–98th percentile to remove outliers. For cell pellet scans, six informative markers were used: four cell-type specific markers (vimentin, HER2, CK19, EGFR), IdU and mitotic pHH3. Cell pellet UMAPs (*k* = 17) were Leiden clustered with resolution 0.2 (code available at 5b_cell_pellet_XRF_analysis.pynb). For breast cancer scans, UMAPs and Leiden clustering were based on the nine markers detectable by XRF (ER, CD44, PR, SMA, HER2, panCK, Ki-67, CK19 and vimentin). Breast cancer UMAPs (*k* = 50) were Leiden clustered with a resolution of 0.5. A ranked *t*-test cluster enrichment analysis was used to assign cell types based on markers enriched in each Leiden cluster. These cluster annotations were projected back onto the cell_matched_mask cell IDs to validate appropriate identities in the spatial domain.

### Marker subcellular localization

To measure marker subcellular localization, nuclear and nonnuclear anndata objects of mean intensities per channel were generated in Steinbock using the nuclei_matched_mask and non-nuclei_matched_mask, respectively. For each cell ID, nuclear to nonnuclear signal to base 2 was used to identify whether a marker was predominantly nuclear (more than 0) or nonnuclear (less than 0).

### IMC

Sample sections on Mylar film after XRF imaging were cut and taped flat onto a glass slide for staining. IMC was conducted using the Fluidigm Hyperion Imaging system using Fluidgm CyTOF IMC software (v.7.0.8493). For direct comparison to XRF, the same Mylar film-mounted gelatin standards and tissue samples imaged by nondestructive XRF were imaged by destructive IMC. For IMC, Mylar film-mounted sections were cut from their acrylic windows and taped flat onto glass slides suitable for Hyperion loading and laser-ablation raster imaging. Samples were raster ablated with a 500-nm step size (200 Hz) to match the 500-nm focal spot used for 500 nm per-pixel XRF. The regions ablated by IMC were raster ablated with two or three rounds of imaging, ensuring that all regions scanned by IMC were completely ablated. Multichannel images with different isotopes in each channel were extracted from the .mcd files output by IMC using imctools v.2.0 (https://github.com/BodenmillerGroup/imctools). The region imaged by XRF was manually identified in IMC scans (Extended Data Figs. 4 and 5). The horizontal dimension of IMC images was scaled 1/1.3 to match XRF images (which were collected at a 40° angle). IMC overview scans matched to the SDD MEZ-XRF images were down-sampled 1/4 to simulate the 2-µm step scans used for MEZ-XRF overview images. For comparisons of IMC ROI to XRF ROI, there was no down-sampling. In all instances, at least a 67% overlap was achieved MEZ-XRF and subsequent IMC images.

### H&E staining

Sample sections on Mylar film after XRF imaging were cut and taped flat onto a glass slide for H&E staining. The glass slide was stained with hematoxylin for 3 min, then washed in deionized water for 1 min. Bluing was performed in Scott’s tap water for 1 min, followed by washing in deionized water for 1 min and in 95% ethanol for 1 min. The slide was then stained with eosin for 1 min, washed with 100% ethanol for 2 min, then with Ultraclear (J.T. Baker, catalog no. 3905.5000PE) for 4 min. The slide was mounted with Eukitt (Sigma, catalog no. 03989), and the bright-field image obtained with a slidescanner (Zeiss, Axio Scan.Z1). The horizontal dimension of H&E images was scaled by 0.9 to match XRF images (which were collected at a 28° angle).

### Image panel generation

All MEX-XRF and IMC single-channel image panels were automatically generated in Python by autoscaling to the 0.5–99.5% gray levels for all pixel intensities across an image row. Code for replicating all image panels in each figure and Extended Data figure is available in the code repository. The multichannel color images (Fig. 6j–l) were assembled from single-channel exports, using the Fiji (v.1.53c)^56^ ‘merge channels’ function, with contrast levels manually adjusted. The final high-resolution IMC image (Fig. 6k) was additionally processed using the scikit-image^57^ Gaussian filter with column shape (sigma = (2.5, 0.2)) to reduce parallel row artifacts.

### Statistics and reproducibility

MEZ-XRF data were collected during three sessions at beamline ID15A, roughly 6 months apart. New samples were stained, and the beamline reassembled to our requirements for each session, where we achieved similar results. Our ability to reconfigure the beamline demonstrates the reproducibility of the MEZ-XRF scanning apparatus. The reproducibility of sample staining was confirmed via IMC during sample staining optimization (not all data shown). For XRF scanning, one field of view, for one section of one sample per cell type was imaged for Figs. 2a–n and 4b–k. One field of view, for one section of one sample was imaged (Figs. 3a–g and 5a–e and Extended Data Figs. 6a–i, 9b–o and 10b–s). A large overview and small ROI scan was collected for one section of one sample per tissue type is presented in Fig. 6. The subsequent IMC images (Fig. 5i–l and Extended Data Figs. 4a–n, 5b–h, 7c,d and 8a) were collected from the same section stained and imaged by XRF in all instances, with the region imaged chosen to be as close as possible to the region imaged using XRF to facilitate comparison.

### Reporting summary

Further information on research design is available in the Nature Portfolio Reporting Summary linked to this article.

## Online content

Any methods, additional references, Nature Portfolio reporting summaries, source data, extended data, supplementary information, acknowledgements, peer review information; details of author contributions and competing interests; and statements of data and code availability are available at 10.1038/s41592-023-01977-x.

## Supplementary information

Supplementary Information Supplementary Tables 1–8.

Reporting Summary

Supplementary Data 1 Design of 3D nylon support for holding Mylar film.

## Data Availability

All XRF, IMC and microscopy raw data files analyzed to generate the presented results are publicly available at Zenodo (10.5281/zenodo.7949102)^51^.

## Code availability

Code for analysis of all XRF, IMC and microscopy data necessary to replicate the results presented in this article are publicly available at https://github.com/BodenmillerGroup/MEZ_XRF. This repository includes notebooks to generate all figure panels presented, except the multicolor panels in Fig. 6 that were manually assembled in Fiji.
