# Supplementary material for: Multielement Z-tag imaging by X-ray fluorescence microscopy for next-generation multiplex imaging
Source: Nat Methods. 2023 Aug 31;20(9):1310–22. doi: 10.1038/s41592-023-01977-x (PMC10482696; doi:10.1038/s41592-023-01977-x)
Supplement: Supplementary file 1 — Supplementary Tables 1–8. [file 41592_2023_1977_MOESM1_ESM.pdf]

nature methods

Article https://doi.org/10.1038/s41592-023-01977-x

Multielement Z-tag imaging by X-ray fluorescence microscopy for next-generation multiplex imaging

In the format provided by the authors and unedited

Supplementary table 1. Scan times for different raster rates.

Scan rate (Hz):	1			
100	100	10,000	10,000	166.67
250	250	62,500	62,500	1,041.67
1,000	1,000	1,000,000	1,000,000	16,666.67
2,500	2,500	6,250,000	6,250,000	104,166.67
Scan rate (Hz):	25			
100	100	10,000	400	6.67
250	250	62,500	2,500	41.67
1,000	1,000	1,000,000	40,000	666.67
2,500	2,500	6,250,000	250,000	4,166.67
Scan rate (Hz):	100			
100	100	10,000	100	1.67
250	250	62,500	625	10.42
1,000	1,000	1,000,000	10,000	166.67
2,500	2,500	6,250,000	62,500	1,041.67
Scan rate (Hz):	250			
100	100	10,000	40	0.67
250	250	62,500	250	4.17
1,000	1,000	1,000,000	4,000	66.67
2,500	2,500	6,250,000	25,000	416.67
Scan rate (Hz):	1,000			
100	100	10,000	10	0.17
250	250	62,500	63	1.04
1,000	1,000	1,000,000	1,000	16.67
2,500	2,500	6,250,000	6,250	104.17
Scan rate (Hz):	2,500			
100	100	10,000	4	0.07
250	250	62,500	25	0.42
1,000	1,000	1,000,000	400	6.67
2,500	2,500	6,250,000	2,500	41.67

Supplementary table 2. Epithelial cell pellet antibody panel. This panel was used for the cell staining in Figure 2.

Tag	Antigen	Clone	Concentration (ug/mL)	Vendor (catalogue #)	Lot #
Y89	Histone H3	HTA28	1.25	Biolegend (641002)	B241679
In113	Histone H3	D1H2	1.25	Cell Signalling Technologies (4499BF)	1
La139	Cytokeratin 8/18	C51	2.5	Cell Signalling Technologies (4546BF)	2
Ce140	Histone H3	HTA28	1.25	Biolegend (641002)	B241679
Pr141	Cytokeratin 5	EP1601Y	5	Abcam (ab214586)	GR299320-I
Nd150	CD44	polyclonal_CD44	1.25	R&D Systems (CFOE0216071)	CFOE0216071
Sm147	Keratin 14 (KRT14)	LL002	0.625	Abcam (ab212547)	GR3366296-1
Eu153	Histone H3	D1H2	0.15	Cell Signalling Technologies (4499BF)	17
Gd158	Vimentin	EPR3776	0.4	Abcam (ab193555)	GR3205290-2
Tb159	c-erbB-2 - Her2	D8F12	2.5	Cell Signalling Technologies (4290)	4
Dy164	Cytokeratin 7	RCK105	5	Abcam (ab9021)	GR3292021-3
Ho165	Ki-67	B56	0.6	BD Biosciences (556003)	8239549
Er170	Cytokeratin 19	A53-B/A2.26	0.32	Sigma Aldrich (319M-1)	GR3333216-1
Tm169	EGFR	EP38Y	1.2	Abcam (ab174481)	GR3271612-1
Yb173	Histone H3	D1H2	0.125	Cell Signalling Technologies (4499BF)	15
Lu175	Keratin Epithelial	AE3	1.25	EMD Millipore (MAB1611)	3255457

Supplementary table 3. Breast cancer antibody panel. This panel was used for the breast cancer tissue staining in Figure 3.

Tag	Antigen	Clone	Concentration (ug/mL)	Vendor (catalogue #)	Lot #
Eu153	Histone H3	HTA28	5	Biolegend (641002)	B200946
Tb159	Cytokeratin 5	EP1601Y	5	Abcam (ab214586)	GR3257526-2
Er170	Cytokeratin 19	A53-B/A2.26	5	Sigma Aldrich (319M-1)	GR3333216-1
Ce140	Estrogen receptor	SP1	5	Abcam (ab187260)	ZF0808
Gd160	CD44	poly_CD44	5	R&D Systems (AF3660)	CFOE0216071
In115	SMA	1A4	5	E-bioscience (14-9760-82)	2183900
Yb173	E- / P-Cadherin	36/E-Cadherin	5	Becton Dickinson (610182)	6251878
Sm152	CD68	KP1	5	E-bioscience (14-0688-82)	2162103
Lu175	pan Cytokeratin	AE1/AE3	5	EMD Millipore (MAB1612) / EMD Millipore (MAB1611)	3252910 / 3255457
Y89	CD3	poly_A0452	5	Agilent (A045201-2)	20073981
La139	Progesterone Receptor A/B	SP2	5	Abcam (ab239793)	1
Pr141	Ki-67	B56	5	BD Biosciences (556003)	8239549
Nd144	c-erbB-2 - Her2	29D8	5	Cell Signalling Technologies (2165)	1
Ho165	Histone H3	D1H2	5	Cell Signalling Technologies (4499BF)	1
Tm169	Vimentin	EPR3776	5	Abcam (ab193555)	GR3238979-10

Supplementary table 4. Antibody panel used for comparing against SABERx2 Z-tag panel. This panel was used for the tissue staining in Extended Data Fig. 7, 9.

Standard Z-tag panel for cell pellet used in Extended Data Fig. 7

Label	Target	Clone	Concentration (ug/mL)	Vendor	Catalogue #	Lot #
Y	pHH3	HTA28	5	Bioledgend	641002	2430398
La	PR	SP2	5	Abcam	ab239793	3252910
Pr	Ki67	B56	5	BD bioscience	556003	8239549
Nd	HER2	29D8	5	Cell Signaling Technologies	2165	1
Tb	CK5	EP1601Y	5	Abcam	ab214586	GR3257526-2
Dy	CK7	RCK105	5	Abcam	ab9021	GR3292021-11
Ho	HH3	D1H2	5	Cell Signaling Technologies	4499BF	1
Tm	Vimentin	EPR3776	5	Abcam	ab193555	GR3238979-10
Lu	PanCK	AE1	5	Millipore	MAB1612	3252910

Standard Z-tag panel for breast tumor used in Extended Data Fig. 9

Label	Target	Clone	Concentration (ug/mL)	Vendor	Catalogue #	Lot #
Y	pHH3	HTA28	5	Bioledgend	641002	2430398
In	SMA	1A4	5	eBioscience	14-9760-82	2382312
La	PR	SP2	5	Abcam	ab239793	3252910
-	ER (primary)	SP1	5	Abcam	ab187260	GR3375050-4
Ce	Rabbit IgG (secondary for ER)	polyclonal_ AI-1000	5	Vecor Labs	AI-1000	ZF0808
Pr	Ki67	B56	5	BD bioscience	556003	8239549
Nd	HER2	29D8	5	Cell Signaling Technologies	2165	1
Sm	CD3	A0452	5	Abcam	ab11089	41261105
Eu	CTLA4	CAL49	5	Abcam	ab251599	GR3344009-12
Gd	PD1	D4W2J	5	Cell Signalling Technology	86163BF	6
Tb	CK5	EP1601Y	5	Abcam	ab214586	GR3257526-2
Dy	CK7	RCK105	5	Abcam	ab9021	GR3292021-11
Ho	HH3	D1H2	5	Cell Signaling Technologies	4499BF	1
Er	CD8	C8/144B	5	eBioscience	14-0085-82	2003078
Tm	Vimentin	EPR3776	5	Abcam	ab193555	GR3238979-10
Yb	CD4	EPR6855	5	Abcam	ab181724	GR3352909-4
Lu	PanCK	AE1	5	Millipore	MAB1612	3252910

Supplementary table 5. SABERx2 panel describing antibodies, bridge DNA IDs, Concatemer ID, Imager IDs, and Imager tags. Left top statement of each panel shows which experiment it was used for.

SABERx2 Z-tag panel for cell pellet used in Fig. 4, Extended Data Fig. 7, 8

Label	Target	Clone	Concentration (ug/mL)	Vendor	Catalogue #	Lot #	bridge ID	1st concatemer ID	2nd concatemer ID	Imager ID	Imager label
La	PR	SP2	2	Abcam	ab239793	GR3352991-6	b.17	b.17*-cc.29	3x(p.29*)-cc.55	i.55	La139
Pr	Ki67	B56	1	BD bioscience	556003	8239549	b.13	b.13*-cc.43	3x(p.43*)-cc.56	i.56	Pr141
Sm	HH3	D1H2	3	Cell Signaling Technologies	4499BF	19	b.18	b.18*-cc.49	3x(p.49*)-cc.33	i.33	Sm152
Gd	HER2	29D8	1	Cell Signaling Technologies	2165	-	b.19	b.19*-cc.50	3x(p.50*)-cc.73	i.73	Gd158
Tb	PanCK	AE1	2	Millipore	MAB1612	3252910	b.22	b.22*-cc.28	3x(p.28*)-cc.25	i.25	Tb159
Dy	CK7	RCK105	1	Abcam	ab9021	GR3380911-1	b.3	b.3*-cc.41	3x(p.41*)-cc.31	i.31	Dy161
Ho	CK5	EP1601Y	2	Abcam	ab214586	GR3334358-3	b.14	b.14*-cc.60	3x(p.60*)-cc.32	i.32	Ho165
Tm	pHH3	HTA28	2	Bioledgend	641002	B241679	b.12	b.12*-cc.42	3x(p.42*)-cc.51	i.51	Tm169
Lu	Vimentin	EPR3776	2	Abcam	ab193555	GR33755150-4	b.7	b.7*-cc.36	3x(p.36*)-cc.52	i.52	Lu175

SABERx2 Z-tag panel for Breast tumor used in Fig. 4, Extended Data Fig. 10

Label	Target	Clone	Concentration (ug/mL)	Vendor	Catalogue #	Lot #	bridge ID	1st concatemer ID	2nd concatemer ID	Imager ID	Imager label
La	PR	SP2	1	Abcam	ab239793	GR3352991-6	b.17	b.17*-cc.29	3x(p.29*)-cc.55	i.55	La139
Ce	ER	SP1	1	Abcam	ab187260	GR3375050-7	b.24	b.24*-cc.37	3x(p.37*)-cc.72	i.72	Ce140
Pr	Ki67	B56	0.5	BD bioscience	556003	8239549	b.13	b.13*-cc.43	3x(p.43*)-cc.56	i.56	Pr141
Nd	pHH3	HTA28	0.5	Bioledgend	641002	B241679	b.12	b.12*-cc.42	3x(p.42*)-cc.51	i.51	Nd148
Sm	CD3	CD3-12	0.5	Abcam	ab11089	GR3256962-4	b.9	b.9*-cc.27	3x(p.27*)-cc.32	i.32	Sm152
Eu	CTLA4	CAL49	0.125	Abcam	ab251599	GR3274686-11	b.6	b.6*-cc.34	3x(p.34*)-cc.53	i.53	Eu153
Gd	PD1	D4W2J	0.5	Cell Signalling Technology	86163BF	4	b.11	b.11*-cc.26	3x(p.26*)-cc.39	i.39	Gd156
Tb	CD4	EPR6855	0.5	Abcam	ab181724	GR3215375-27	b.5	b.5*-cc.47	3x(p.47*)-cc.70	i.70	Tb159
Dy	CK7	RCK105	0.5	Abcam	ab9021	GR3380911-1	b.3	b.3*-cc.41	3x(p.41*)-cc.31	i.31	Dy161
Ho	HH3	D1H2	3	Cell Signaling Technologies	4499BF	19	b.18	b.18*-cc.49	-	i.49	Ho165
Er	CD8	C8/144B	1	eBioscience	14-0085-82	2132595	b.1	b.1*-cc.30	3x(p.30*)-cc.38	i.38	Er166
Tm	HER2	29D8	0.5	Cell Signaling Technologies	2165	-	b.19	b.19*-cc.50	3x(p.50*)-cc.73	i.73	Tm169
Lu	PanCK	AE1	2	Millipore	MAB1612	3252910	b.22	b.22*-cc.28	3x(p.28*)-cc.25	i.25	Lu175

SABERx2 Z-tag panel for Breast tumor used in Fig. 6

Label	Target	Clone	Concentration (ug/mL)	Vendor	Catalogue #	Lot #	bridge ID	1st concatemer ID	2nd concatemer ID	Imager ID	Imager label
La	CD4	EPR6855	0.5	Abcam	ab181724	GR3215375-27	b.5	b.5*-cc.29	3x(p.29*)-cc.55	i.55	La139
Ce	ER	SP1	0.5	Abcam	ab187260	GR3375050-7	b.24	b.24*-cc.37	3x(p.37*)-cc.72	i.72	Ce140
Pr	Ki67	B56	0.5	BD bioscience	556003	8239549	b.13	b.13*-cc.43	3x(p.43*)-cc.56	i.56	Pr141
Nd	pHH3	HTA28	0.5	Bioledgend	641002	B241679	b.12	b.12*-cc.42	3x(p.42*)-cc.51	i.51	Nd148
Sm	CD3	CD3-12	0.5	Abcam	ab11089	GR3256962-4	b.9	b.9*-cc.27	3x(p.27*)-cc.32	i.32	Sm152
Tb	CD11c	EP1347Y	0.1	Abcam	ab216655	GR3210349-11	b.3	b.3*-cc.41	3x(p.41*)-cc.31	i.31	Tb159
Ho	HH3	D1H2	2	Cell Signaling Technologies	4499BF	19	b.18	b.18*-cc.49	-	i.49	Ho165
Er	CD8	C8/144B	1	eBioscience	14-0085-82	2132595	b.1	b.1*-cc.30	3x(p.30*)-cc.38	i.38	Er166
Tm	HER2	29D8	0.5	Cell Signaling Technologies	2165	-	b.19	b.19*-cc.50	3x(p.50*)-cc.73	i.73	Tm169
Lu	PanCK	AE1	1	Millipore	MAB1612	3252910	b.22	b.22*-cc.28	3x(p.28*)-cc.25	i.25	Lu175

SABERx2 Z-tag panel for Appendix used in Fig. 6

Label	Target	Clone	Concentration (ug/mL)	Vendor	Catalogue #	Lot #	bridge ID	1st concatemer ID	2nd concatemer ID	Imager ID	Imager label
Pr	Ki67	B56	0.5	BD bioscience	556003	8239549	b.13	b.13*-cc.43	3x(p.43*)-cc.56	i.56	Pr141
Nd	pHH3	HTA28	0.5	Bioledgend	641002	B241679	b.12	b.12*-cc.42	3x(p.42*)-cc.51	i.51	Nd148
Sm	CD3	CD3-12	0.5	Abcam	ab11089	GR3256962-4	b.9	b.9*-cc.27	3x(p.27*)-cc.32	i.32	Sm152
Eu	CTLA4	CAL49	0.1	Abcam	ab251599	GR3274686-11	b.6	b.6*-cc.34	3x(p.34*)-cc.53	i.53	Eu153
Gd	PD1	D4W2J	0.5	Cell Signalling Technology	86163BF	4	b.11	b.11*-cc.26	3x(p.26*)-cc.39	i.39	Gd155
Tb	CD20	L26	0.5	Invitrogen	14-0202-82	2059976	b.2	b.2*-cc.25	3x(p.25*)-cc.31	i.31	Tb159
Ho	HH3	D1H2	3	Cell Signaling Technologies	4499BF	19	b.18	b.18*-cc.49	-	i.49	Ho165
Er	CD8a	C8/144B	0.5	eBioscience	14-0085-82	2132595	b.1	b.1*-cc.30	3x(p.30*)-cc.38	i.38	Er166
Tm	CD4	EPR6855	0.5	Abcam	ab181724	GR3215375-27	b.5	b.5*-cc.29	3x(p.29*)-cc.55	i.55	Yb171
Lu	CD11c	EP1347Y	0.1	Abcam	ab216655	GR3210349-11	b.3	b.3*-cc.41	3x(p.41*)-cc.37	i.37	Lu175

Supplementary table 6. DNA sequence for each barcode ID.

1st Concatemer: Insert "TT" as a spacer between bridge and primer sequence
2nd Concatemer: Insert "T" for each of three primer* repeats
2nd Concatemer: Insert "TTT" between three primer* repeats and new primer.

Bridge DNA		Primer for PER extention			
ID	Sequence	ID	Sequence	ID	Sequence
b.1	ATTATCCCTACCGCCAAATCTCCGTGTCCTTAACCGACCTAT	b.1*	ATAGGTCGGTTAAGGACACGGAGATTTGGCGGTAGGGATAAT	p.25	CCAATAATA
b.2	ACCTCTTACCGCATATACCGACCCAGTAGTATGTATCGAGCT	b.2*	AGCTCGATACATACTACTGGGTCGGTATATGCGGTAAGAGGT	p.26	ATAAACCTA
b.3	CTTTGTATCCCCGCATTGTCAACCGTGATATGCGCTTTAACT	b.3*	AGTTAAAGCGCATATCACGGTTGACAATGCGGGGATACAAAG	p.27	CATCATCAT
b.5	CAAGTATCTCCTTCGCCCTATTCGCCATAATTTCCACCCCGA	b.5*	TCGGGGTGGAAATTATGGCGAATAGGGCGAAGGAGATACTTG	p.28	CAACTTAAC
b.6	CAAGTATCTCCTTCGCCCTATTCGCCATAATTTCCACCCCGA	b.6*	TCGGGGTGGAAATTATGGCGAATAGGGCGAAGGAGATACTTG	p.29	TCTAAAATC
b.7	CAAGTATCTCCTTCGCCCTATTCGCCATAATTTCCACCCCGA	b.7*	TCGGGGTGGAAATTATGGCGAATAGGGCGAAGGAGATACTTG	p.30	AATACTCTC
b.9	CAAGTATCTCCTTCGCCCTATTCGCCATAATTTCCACCCCGA	b.9*	TCGGGGTGGAAATTATGGCGAATAGGGCGAAGGAGATACTTG	p.31	TTATTCACT
b.11	ATTATCCCTACCGCCAAATCTCCGTGTCCTTAACCGACCTAT	b.11*	ATAGGTCGGTTAAGGACACGGAGATTTGGCGGTAGGGATAAT	p.32	CTTTTTTTC
b.12	ATTATCCCTACCGCCAAATCTCCGTGTCCTTAACCGACCTAT	b.12*	ATAGGTCGGTTAAGGACACGGAGATTTGGCGGTAGGGATAAT	p.33	CCTTCTATT
b.13	ATTATCCCTACCGCCAAATCTCCGTGTCCTTAACCGACCTAT	b.13*	ATAGGTCGGTTAAGGACACGGAGATTTGGCGGTAGGGATAAT	p.34	CTCTACTAC
b.14	ATTATCCCTACCGCCAAATCTCCGTGTCCTTAACCGACCTAT	b.14*	ATAGGTCGGTTAAGGACACGGAGATTTGGCGGTAGGGATAAT	p.36	AACTAATCT
b.15	GTTTCAAGAGTCCGTCGCAAATTCCACTACACGCTACGCCCA	b.15*	TGGGCGTAGCGTGTAGTGGAATTTGCGACGGACTCTTGAAAC	p.37	TTTCTCTTC
b.17	GTTTAGCTTTTCACGTCGCCAATAGGTCCTTCCGTACACCAC	b.17*	GTGGTGTACGGAAGGACCTATTGGCGACGTGAAAAGCTAAAC	p.38	AACATACTA
b.18	GGTTCACATTATTCGCGCTTTACTGATACAAGACCTCACGCT	b.18*	AGCGTGAGGTCTTGTATCAGTAAAGCGCGAATAATGTGAACC	p.39	TTCATTTAC
b.19	ACCTCTTACCGCATATACCGACCCAGTAGTATGTATCGAGCT	b.19*	AGCTCGATACATACTACTGGGTCGGTATATGCGGTAAGAGGT	p.41	CAATCAAAA
b.22	GTGTATTGTCTCGCGTATAGTCACCTGTTAGACGTCGGTGGT	b.22*	ACCACCGACGTCTAACAGGTGACTATACGCGAGACAATACAC	p.42	CTTACAAAC
b.24	TATGCAGAGTCGTGTATTTCCTTTGCGCACGTACTCAATTCT	b.24*	AGAATTGAGTACGTGCGCAAAGGAAATACACGACTCTGCATA	p.43	ACAAATAAC
				p.45	CCCTTATTT
				p.47	TTCTTACTC
				p.49	CATTTATCC
				p.50	ATACTTCAC
				p.51	TACCTCTAA
				p.52	CTCCTATTT
				p.53	CTATCCAAA
				p.55	TCATTACTT
				p.56	CTAAATCTC
				p.60	ACTAACTAT
				p.69	TTTCTATCT
				p.70	TCCTTTTAT
				p.72	CATATTACA
				p.73	TTCCTAATC

Hairpin for PER extention		Imager	
ID	Sequence	ID	Sequence
h.25.25	ACCAATAATAGGGCCTTTTGGCCCTATTATTGGTTATTATTGG/3InvdT/	i.25	TTTATTATTGGTTATTATTGGT
h.26.26	AATAAACCTAGGGCCTTTTGGCCCTAGGTTTATTTAGGTTTAT/3InvdT/	i.26	TTTAGGTTTATTTAGGTTTATT
h.27.27	ACATCATCATGGGCCTTTTGGCCCATGATGATGTATGATGATG/3InvdT/	i.27	TTATGATGATGTATGATGATGT
h.28.28	ACAACTTAACGGGCCTTTTGGCCCGTTAAGTTGTGTTAAGTTG/3InvdT/	i.28	TTGTTAAGTTGTGTTAAGTTGT
h.29.29	ATCTAAAATCGGGCCTTTTGGCCCGATTTTAGATGATTTTAGA/3InvdT/	i.29	TTGATTTTAGATGATTTTAGAT
h.30.30	AAATACTCTCGGGCCTTTTGGCCCGAGAGTATTTGAGAGTATT/3InvdT/	i.30	TTGAGAGTATTTGAGAGTATTT
h.31.31	ATTATTCACTGGGCCTTTTGGCCCAGTGAATAATAGTGAATAA/3InvdT/	i.31	TTAGTGAATAATAGTGAATAAT
h.32.32	ACTTTTTTTCGGGCCTTTTGGCCCGAAAAAAAGTGAAAAAAAG/3InvdT/	i.32	TTGAAAAAAAGTGAAAAAAAGT
h.33.33	ACCTTCTATTGGGCCTTTTGGCCCAATAGAAGGTAATAGAAGG/3InvdT/	i.33	TTAATAGAAGGTAATAGAAGGT
h.34.34	ACTCTACTACGGGCCTTTTGGCCCGTAGTAGAGTGTAGTAGAG/3InvdT/	i.34	TTGTAGTAGAGTGTAGTAGAGT
h.36.36	AAACTAATCTGGGCCTTTTGGCCCAGATTAGTTTAGATTAGTT/3InvdT/	i.36	TTAGATTAGTTTAGATTAGTTT
h.37.37	ATTTCTCTTCGGGCCTTTTGGCCCGAAGAGAAATGAAGAGAAA/3InvdT/	i.37	TTGAAGAGAAATGAAGAGAAAT
h.38.38	AAACATACTAGGGCCTTTTGGCCCTAGTATGTTTTAGTATGTT/3InvdT/	i.38	TTTAGTATGTTTTAGTATGTTT
h.39.39	ATTCATTTACGGGCCTTTTGGCCCGTAAATGAATGTAAATGAA/3InvdT/	i.39	TTGTAAATGAATGTAAATGAAT
h.41.41	ACAATCAAAAGGGCCTTTTGGCCCTTTTGATTGTTTTTGATTG/3InvdT/	i.41	TTTTTTGATTGTTTTTGATTGT
h.42.42	ACTTACAAACGGGCCTTTTGGCCCGTTTGTAAGTGTTTGTAAG/3InvdT/	i.42	TTGTTTGTAAGTGTTTGTAAGT
h.43.43	AACAAATAACGGGCCTTTTGGCCCGTTATTTGTTGTTATTTGT/3InvdT/	i.43	TTGTTATTTGTTGTTATTTGTT
h.45.45	ACCCTTATTTGGGCCTTTTGGCCCAAATAAGGGTAAATAAGGG/3InvdT/	i.45	TTAAATAAGGGTAAATAAGGGT
h.47.47	ATTCTTACTCGGGCCTTTTGGCCCGAGTAAGAATGAGTAAGAA/3InvdT/	i.47	TTGAGTAAGAATGAGTAAGAAT
h.49.49	ACATTTATCCGGGCCTTTTGGCCCGGATAAATGTGGATAAATG/3InvdT/	i.49	TTGGATAAATGTGGATAAATGT
h.50.50	AATACTTCACGGGCCTTTTGGCCCGTGAAGTATTGTGAAGTAT/3InvdT/	i.50	TTGTGAAGTATTGTGAAGTATT
h.51.51	ATACCTCTAAGGGCCTTTTGGCCCTTAGAGGTATTTAGAGGTA/3InvdT/	i.51	TTTTAGAGGTATTTAGAGGTAT
h.52.52	ACTCCTATTTGGGCCTTTTGGCCCAAATAGGAGTAAATAGGAG/3InvdT/	i.52	TTAAATAGGAGTAAATAGGAGT
h.53.53	ACTATCCAAAGGGCCTTTTGGCCCTTTGGATAGTTTTGGATAG/3InvdT/	i.53	TTTTTGGATAGTTTTGGATAGT
h.55.55	ATCATTACTTGGGCCTTTTGGCCCAAGTAATGATAAGTAATGA/3InvdT/	i.55	TTAAGTAATGATAAGTAATGAT
h.56.56	ACTAAATCTCGGGCCTTTTGGCCCGAGATTTAGTGAGATTTAG/3InvdT/	i.56	TTGAGATTTAGTGAGATTTAGT
h.60.60	AACTAACTATGGGCCTTTTGGCCCATAGTTAGTTATAGTTAGT/3InvdT/	i.60	TTATAGTTAGTTATAGTTAGTT
h.69.69	ATTTCTATCTGGGCCTTTTGGCCCAGATAGAAATAGATAGAAA/3InvdT/	i.69	TTAGATAGAAATAGATAGAAAT
h.70.70	ATCCTTTTATGGGCCTTTTGGCCCATAAAAGGATATAAAAGGA/3InvdT/	i.70	TTATAAAAGGATATAAAAGGAT
h.72.72	ACATATTACAGGGCCTTTTGGCCCTGTAATATGTTGTAATATG/3InvdT/	i.72	TTTGTAATATGTTGTAATATGT
h.73.73	ATTCCTAATCGGGCCTTTTGGCCCGATTAGGAATGATTAGGAA/3InvdT/	i.73	TTGATTAGGAATGATTAGGAAT

Supplementary table 7. Primer exchange reaction condition for synthesizing concatemers.

Concatemer ID	Hairpin concentration (uM)	temperature	Hour
b.12*-cc.42	0.5	37	24
b.17*-cc.29	1	37	24
b.13*-cc.43	0.25	37	3
b.19*-cc.50	1	37	3
b.14*-cc.60	2	37	3
b.3*-cc.41	0.5	37	24
b.18*-cc.49	1	37	3
b.7*-cc.36	0.2	37	24
b.22*-cc.28	0.5	37	3
b.24*-cc.37	1	37	24
b.9*-cc.27	0.5	37	3
b.6*-cc.34	0.5	37	24
b.11*-cc.26	1	37	3
b.5*-cc.47	1	37	3
b.1*-cc.30	0.5	37	24
b.15*-cc.45	0.5	37	24
b.2*-cc.25	0.5	37	3
3x(p.29*)-cc.55	0.4	37	3
3x(p.43*)-cc.56	0.2	37	3
3x(p.41*)-cc.31	0.25	37	3
3x(p.28*)-cc.25	0.25	37	3
3x(p.37*)-cc.72	0.5	25	24
3x(p.42*)-cc.51	1	25	24
3x(p.27*)-cc.32	1	37	3
3x(p.34*)-cc.53	1	25	24
3x(p.26*)-cc.39	0.2	37	3
3x(p.47*)-cc.70	0.5	25	20
3x(p.30*)-cc.38	0.5	37	1.5
3x(p.50*)-cc.73	0.25	37	3
3x(p.49*)-cc.33	0.5	25	24
3x(p.60*)-cc.32	1	37	3
3x(p.36*)-cc.52	1	25	24
3x(p.45*)-cc.69	1	37	3
3x(p.25*)-cc.31	0.2	37	3

Supplementary table 8. Software package versions used during Python processing.

Software	Version
Python	3.8.8
Conda	4.10.1
Numpy	1.20
imctools	2.1.7
Scanpy	1.7.2
Anndata	0.7.5
SciPy	1.6.2
Matplotlib	3.4.1
Pandas	1.2.3
PyMCA	5.6.3
Seaborn	0.11.1
h5py	2.10
Steinbock	0.5.2
xarray	0.17.0
Deepcell	0.9.0
