# Supplementary material for: Multielement Z-tag imaging by X-ray fluorescence microscopy for next-generation multiplex imaging
Source: Nat Methods. 2023 Aug 31;20(9):1310–22. doi: 10.1038/s41592-023-01977-x (PMC10482696; doi:10.1038/s41592-023-01977-x)
Supplement: Supplementary file 2 — Reporting Summary [file 41592_2023_1977_MOESM2_ESM.pdf]

nature research

Corresponding author(s): Bernd Bodenmiller

Last updated by author(s): Jun 7, 2023

Reporting Summary

Nature Research wishes to improve the reproducibility of the work that we publish. This form provides structure for consistency and transparency in reporting. For further information on Nature Research policies, see our Editorial Policies and the Editorial Policy Checklist.

Statistics

For all statistical analyses, confirm that the following items are present in the figure legend, table legend, main text, or Methods section.

n/a	Confirmed	
☐	☒	The exact sample size (*n*) for each experimental group/condition, given as a discrete number and unit of measurement
☐	☒	A statement on whether measurements were taken from distinct samples or whether the same sample was measured repeatedly
☒	☐	The statistical test(s) used AND whether they are one- or two-sided<br>*Only common tests should be described solely by name; describe more complex techniques in the Methods section.*
☒	☐	A description of all covariates tested
☒	☐	A description of any assumptions or corrections, such as tests of normality and adjustment for multiple comparisons
☐	☒	A full description of the statistical parameters including central tendency (e.g. means) or other basic estimates (e.g. regression coefficient) AND variation (e.g. standard deviation) or associated estimates of uncertainty (e.g. confidence intervals)
☒	☐	For null hypothesis testing, the test statistic (e.g. *F*, *t*, *r*) with confidence intervals, effect sizes, degrees of freedom and *P* value noted<br>*Give P values as exact values whenever suitable.*
☒	☐	For Bayesian analysis, information on the choice of priors and Markov chain Monte Carlo settings
☒	☐	For hierarchical and complex designs, identification of the appropriate level for tests and full reporting of outcomes
☐	☒	Estimates of effect sizes (e.g. Cohen's *d*, Pearson's *r*), indicating how they were calculated

Our web collection on statistics for biologists contains articles on many of the points above.

Software and code

Policy information about availability of computer code

Data collection	IMC data collected with Fluidgm CyTOF imaging mass cytometry software (7.0.8493).
Data analysis	All analysis conducted in Python (V 3.8.8) using freely available Python packages. Key packages and version numbers include Conda (4.10.1), Numpy (1.20), imctools (2.1.7), Scanpy (1.7.2), Anndata (0.7.5), SciPy (1.6.2), Matplotlib (3.4.1), Pandas (1.2.3), PyMCA (5.6.3), Seaborn (0.11.1), h5py (2.10), Steinbock (0.5.2), xarray (0.17.0), Deepcell (0.9.0).Jupyter notebooks to go from raw data to the generated figures in the paper are available at https://github.com/BodenmillerGroup/MEZ-XRF

For manuscripts utilizing custom algorithms or software that are central to the research but not yet described in published literature, software must be made available to editors and reviewers. We strongly encourage code deposition in a community repository (e.g. GitHub). See the Nature Research guidelines for submitting code & software for further information.

Data

Policy information about availability of data

All manuscripts must include a data availability statement. This statement should provide the following information, where applicable:

- Accession codes, unique identifiers, or web links for publicly available datasets
- A list of figures that have associated raw data
- A description of any restrictions on data availability

All XRF, IMC and microscopy raw data files analysed to generate the presented results are publicly available at Zenodo (DOI: 10.5281/zenodo.7949102)

Field-specific reporting

Please select the one below that is the best fit for your research. If you are not sure, read the appropriate sections before making your selection.

☒ Life sciences ☐ Behavioural & social sciences ☐ Ecological, evolutionary & environmental sciences

For a reference copy of the document with all sections, see nature.com/documents/nr-reporting-summary-flat.pdf

Life sciences study design

All studies must disclose on these points even when the disclosure is negative.

Sample size	For each iteration of MEZ-XRF, a single scan was collected per cell/tissue type. The multiple cell/tissue types imaged per iteration demonstrated the reproducibility of MEZ-XRF. We did not collect more scans of multiple replicates for each sample type as beamtime was limited and we prioritised collected a wider variety of samples (rather than replicates of the same samples) to demonstrate the versatility of MEZ-XRF. This is particularly relevant as our study represents methods development rather than answering a particular biological question.
Data exclusions	Complete raw data provided for all presented results. No scans were excluded.
Replication	MEZ-XRF data was collected across 3 different visits to beamline ID-15A, ~6 months apart. New samples were stained, and the beamline reassembled to our requirements for each visit, where we achieved similar results. All visits were successful. Our ability to reconfigure the beamline across visits demonstrates the reproducibility of the MEZ-XRF scanning apparatus. The reproducibility of sample staining was confirmed via IMC during sample staining optimisation (not all data shown).
Randomization	Randomization was not applicable for this experiment as samples did not need to be randomly allocated to different groups. Our study is the development of a method rather than comparison of biological features between groups.
Blinding	Blinding was not used for this experiment as the molecular markers revealed through MEZ-XRF and IMC scanning revealed exactly which of the samples were being imaged/analysed. Our study is a method development study rather than a biological study.

Reporting for specific materials, systems and methods

We require information from authors about some types of materials, experimental systems and methods used in many studies. Here, indicate whether each material, system or method listed is relevant to your study. If you are not sure if a list item applies to your research, read the appropriate section before selecting a response.

Materials & experimental systems

n/a	Involved in the study	
☐	☒	Antibodies
☐	☒	Eukaryotic cell lines
☒	☐	Palaeontology and archaeology
☒	☐	Animals and other organisms
☒	☐	Human research participants
☒	☐	Clinical data
☒	☐	Dual use research of concern

Methods

n/a	Involved in the study	
☒	☐	ChIP-seq
☒	☐	Flow cytometry
☒	☐	MRI-based neuroimaging

Antibodies

Antibodies used	For Figures 2-3, Supplementary Table 1 & 2 list antibodies used. For the SABER amplification panel in Figure 4-5, for clarity, antibodies are detailed in Supplementary File 2 alongside additional SABER tag information (i.e. DNA oligo sequences) needed to construct SABER reagents.
Validation	All antibodies used in our manuscript were previously validated by our group (Jackson, H. W. et al. The single-cell pathology landscape of breast cancer. Nature 1–6 (2020). doi:10.1038/s41586-019-1876-x). This validation included immunofluorescence imaging with single antibodies prior to metal conjugation, as well as cell type and subcellular location specificity testing in positive and negative control tissues.

Eukaryotic cell lines

Policy information about cell lines

Cell line source(s)	ZR-75-1 (CRL-1500), MCF10a (CRL-10317), SKBR3 (HTB-30), and A431 (CRL-1555) cells lines were sourced from the American Type Culture Collection (ATCC).

April 2020

Authentication	Cell lines were derived from ATCC vials. Figure 2-3 did confirm these cell lines expressed their expected markers. No further authentication work was conducted.
Mycoplasma contamination	Cell lines were derived from ATCC vials but were not further authenticated. They were not tested for mycoplasma contamination.
Commonly misidentified lines (See ICLAC register)	No commonly misidentified lines were used.
